# Anthropogenic activities mediate stratification and stability of microbial communities in freshwater sediments

**DOI:** 10.1186/s40168-023-01612-z

**Published:** 2023-08-26

**Authors:** Xiaotian Zhou, Jay T. Lennon, Xiang Lu, Aidong Ruan

**Affiliations:** 1 The National Key Laboratory of Water Disaster Prevention, Hohai University, Nanjing, 210024 China; 2 College of Hydrology and Water Resources, Hohai University, Nanjing, 210024 China; 3 Department of Biology, Indiana University, Bloomington, IN 47405 USA

## Abstract

**Background:**

Freshwater sediment microbes are crucial decomposers that play a key role in regulating biogeochemical cycles and greenhouse gas emissions. They often exhibit a highly ordered structure along depth profiles. This stratification not only reflects redox effects but also provides valuable insights into historical transitions, as sediments serve as important archives for tracing environmental history. The Anthropocene, a candidate geological epoch, has recently garnered significant attention. However, the human impact on sediment zonation under the cover of natural redox niches remains poorly understood. Dam construction stands as one of the most far-reaching anthropogenic modifications of aquatic ecosystems. Here we attempted to identify the ecological imprint of damming on freshwater sediment microbiome.

**Results:**

We conducted a year-round survey on the sediment profiles of Lake Chaohu, a large shallow lake in China. Through depth-discrete shotgun metagenomics, metataxonomics, and geophysiochemical analyses, we unveiled a unique prokaryotic hierarchy shaped by the interplay of redox regime and historical damming (labeled by the ^137^Cs peak in AD 1963). Dam-induced initial differentiation was further amplified by nitrogen and methane metabolism, forming an abrupt transition governing nitrate–methane metabolic interaction and gaseous methane sequestration depth. Using a random forest algorithm, we identified damming-sensitive taxa that possess distinctive metabolic strategies, including energy-saving mechanisms, unique motility behavior, and deep-environment preferences. Moreover, null model analysis showed that damming altered microbial community assembly, from a selection-oriented deterministic process above to a more stochastic, dispersal-limited one below. Temporal investigation unveiled the rapid transition zone as an ecotone, characterized by high species richness, low community stability, and emergent stochasticity. Path analysis revealed the observed emergent stochasticity primarily came from the high metabolic flexibility, which potentially contributed to both ecological and statistical neutralities.

**Conclusions:**

We delineate a picture in which dam-induced modifications in nutrient availability and sedimentation rates impact microbial metabolic activities and generate great changes in the community structure, assembly, and stability of the freshwater sediment microbiome. These findings reflect profound ecological and biogeochemical ramifications of human–Earth system interactions and help re-examine the mainstream views on the formation of sediment microbial stratification.

Video Abstract

**Supplementary Information:**

The online version contains supplementary material available at 10.1186/s40168-023-01612-z.

## Background

In aquatic habitats, microbial communities in sediments play a crucial role in regulating nutrient cycle and energy flow ranging from small ponds to the global ocean. Many of them are highly structured with depth and exhibit a nonuniform turnover, which coincides with geochemical transition zones (GTZs) [1]. Microbial communities exhibit changes that reflect the oxic–anoxic transition zone (OATZ) [2], the nitrate–ammonium transition zone (NATZ) [3], the nitrate/nitrite-methane transition zone (NMTZ) [4, 5], and the most well-known sulfate–methane transition zone (SMTZ) [6]. Characterizing the burial depth of GTZs is important for estimating greenhouse gas emissions as well as enhancing the understanding of subfloor biogeochemical cycles [7].

A consensus is that these stratifications and orders are primarily governed by redox chemistry. Resembling the Winogradsky column [8], energy availability drives the hierarchy of electron acceptors [9–11]. However, the diverse patterns of sediment stratification suggest the existence of other potential drivers beyond redox chemistry. In marine systems, redox cascades can be disrupted by hydrodynamic disturbance [12] or benthic bioturbation [13], or vary with water depth [14], eutrophication, and sedimentation rates [7, 11]. In comparison, few studies have clearly discussed these effects on redox zonation in freshwater sediments. Differences between the two systems make it challenging to apply marine-derived models to freshwater sediments [15]. Freshwater systems typically have lower dissolved sulfate levels, where sulfur cycling is considered relatively minor [16]; whether the SMTZ well established in marine systems is prevalent in freshwater sediments and whether it would be replaced by NMTZ remain to be explored. Another difference is the degree of human impact: the higher wetted perimeter of lakes and closer ties with human activities make it a nontrivial factor in shaping freshwater sediment structure.

Dam construction, surging in the mid-twentieth century, is considered one of the most far-reaching anthropogenic modifications of aquatic ecosystems [17, 18]. Unlike episodic sedimentation caused by floods or extreme weather, these management strategies could induce hidden inherited changes known as legacy effects. It may bring priority effects on microbial community assemblages and generate alternative successional trajectories during burial [19–21]. Coupled with enhanced fertilizer and wastewater inputs, it may also permanently alter sediment biogeochemical properties by accelerating N/P retention [22]. This shift is likely to result in nitrate becoming the primary electron acceptor for anaerobic methane oxidation (AOM). However, previous studies on the damming impact on lake microbial ecosystems have mostly overlooked the vertical response of sediment microbiomes, limiting our understanding of stratified changes and their relationship with redox cascades.

Understanding community assembly processes and stratification formation requires depicting community stability patterns. Steep transitions in sediments closely fit the ecotone model, wherein species richness often tends to peak and the local communities are highly dynamic and unstable over time [23, 24]. Community stability can be reflected in both the multi-timepoint dissimilarity and the holistic flexibility of microbes to adapt to different environments. The former can be directly obtained by temporal investigations; community dynamic processes being rhythmic or chaotic, deterministic or stochastic, largely depend on the observational time scale. The latter can be further manifested as cell chemotaxis and niche breadth. Chemotaxis, by which cells sense chemical gradients and move directionally with preference, reflects spatiotemporal heterogeneity of energy supply; those living in stable, homogenous environments harbor fewer methyl-accepting chemotaxis proteins (MCPs) and response regulatory proteins [25, 26]. On the other hand, in a niche-based community, higher metabolic flexibility indicates broader niche breadth, as variations in resource availability select flexible habitat generalists rather than niche-restricted specialists [27]. To date, the link between stratification and stability in sediment microbiomes remains poorly established.

**Fig. 1:**
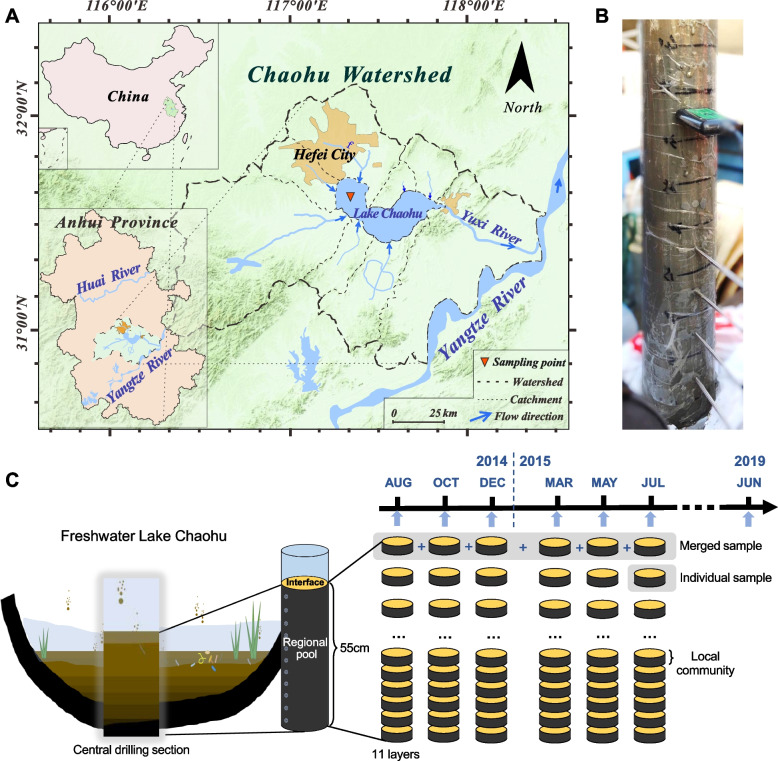
Sampling site and strategy.** A** Location of the sampling site and map of the Chaohu Basin, China. **B** Present physicochemical parameters of each sediment interval were determined in situ on cores without destroying the connectivity between layers. **C** Schematic plot of the spatiotemporal sampling strategy

In this study, we ask about the role of anthropogenic activities in forming biogeochemical stratification in freshwater lake sediments. We attempted to identify the legacy effect of dam construction with emphasis on (i) microbial taxonomic and functional stratification, (ii) the coupling effect with classic redox cascade, (iii) community assembly processes, and (iv) community stability. We undertook this study with a 1-year sampling strategy in Lake Chaohu, a major freshwater lake in China (Fig. 1). Chaohu Dam was constructed in 1962 which triggered eutrophication in the 1970s [28] (see Text S1 for details). We hypothesized (i) damming would disrupt the regular redox order, leading to rapid stratification through changes in energy inputs, sedimentation properties, and community assembly processes; (ii) the NMTZ would replace the SMTZ with N-dependent AOM as the key factor controlling upward methane flux, and (iii) significant energy differences would occur at the rapid stratification zone and enhance local community fluctuations. To test these hypotheses, we identified microbial taxonomic and functional stratifications with combination of shotgun metagenomics and amplicon sequencing approaches and proposed a potential past–present coupling mechanism for stratification by elucidating the processes of sedimentation and microbial community dynamics.

## Methods

### Study site and sampling procedures

Consistent with previous chronological studies of Chaohu sediments [28, 29], the sampling site was set in the central part of western Chaohu (31° 37′ 23″ N, 117° 22′ 21″ E, Fig. 1A), where the lake is more eutrophic and away from the nearshore hydraulic disturbance zone. We sampled bimonthly from August 2014 to July 2015, which provided seasonal snapshots for the sediment profile (Fig. 1B–C). On June 2 2019, we performed additional sampling for gaseous methane estimation. Sediment cores were collected using a gravity corer outfitted with a clear polycarbonate tube (inner diameter of 8.2 cm, length of 60 cm). Considering the destructive nature of sediment sampling, we controlled the spatial sampling-site bias within a 100 m-distance for seasonal samples. An excision of a 1-cm-thick margin section was applied for each 5-cm-interval subsample to avoid sidewall contamination and minimize margin effects. Samples were debris-trimmed and zoobenthos-removed and then transferred into a clean sealed sample bag and briefly homogenized by manual manipulation. These homogenized samples were frozen in liquid nitrogen and stored at − 80 °C for subsequent analysis.

### Environmental parameters

The physicochemical properties of the sediments were measured immediately after lifting sediment cores off the water surface to maintain the integrity of the core structure (Fig. 1B). In situ sediment temperature was obtained using an electric thermometer. Volumetric water content (${\mathrm{Moi}}_{(v)}$) and conductivity (Cond) were measured via a portable soil moisture and EC sensor (TR-6D, Shunkeda, Beijing, China). Redox potential (ORP) and pH were measured using Unisense redox (metal tip, standard hydrogen potential) and pH microelectrodes (Unisense, Aarhus, Denmark). In the laboratory, total organic carbon (TOC) was determined using an organic carbon analyzer (Multi N/C 2100 TOC, Analytik AG, Jena, Germany) after pretreating samples with 1 mol^−1^ HCl to remove inorganic carbon. Sediment grain size was analyzed by a laser diffraction particle size analyzer (LS 13320, Beckman Coulter, USA, measuring range: 0.017 ~ 2000 μm). We calculated the median diameter (*D*_50_) and mean grain size (MGS) to represent the particle size characteristics. We identified the texture class of the sediments according to the soil textural triangle [30].

### Sediment interstitial space feature and methane accumulation

In sediments, methane (CH_4_) supersaturation occurs and forms bubbles. Previous studies have shown that CH_4_ acts as the main component of sediment gas space [31–33]. Based on this, we used the gas space as a proxy for the CH_4_ accumulation degree (ebullitive CH_4_ flux). The gas space volume percent (${\mathrm{VP}}_{(a)}$) was calculated based on the measured volumetric and mass water contents at each specific depth, which is rigorously physics-oriented [32]. In brief, the total interstitial space volume percent (TIS) consists of the gas space volume percent ${\mathrm{VP}}_{(a)}$ and the volumetric water content ${\mathrm{Moi}}_{(v)}$:

1 $$\mathrm{TIS}={\mathrm{Moi}}_{(v)}+{\mathrm{VP}}_{(a)}$$

The physical meaning of volumetric water content is as follows:

2 $${\mathrm{Moi}}_{(v)}=\frac{{V}_{(w)}}{{V}_{(T)}}=\frac{{V}_{(w)}}{{V}_{(s)}+{V}_{(w)}+{V}_{(a)}}$$

where ${V}_{(w)}$ is the volume of the pore water, ${V}_{(s)}$ is the volume of the solid particles, ${V}_{(a)}$ is the volume of the gas space, and ${V}_{(T)}$ represents the total volume of the sediment layer sample.

Measured through the drying method in lab, the mass water content ${\mathrm{Moi}}_{(m)}$ is calculated as:

3 $${\mathrm{Moi}}_{(m)}=\frac{{M}_{(w)}}{{M}_{(T)}}=\frac{{M}_{(w)}}{{M}_{(s)}+{M}_{(w)}}$$

where ${M}_{(w)}$ is the mass of the sediment water, ${M}_{(s)}$ is the mass of solids, and ${M}_{(T)}$ is the total mass of the sediment layer sample.

As described in our previous study [32], the layered gas space volume percent ${\mathrm{VP}}_{(a)}$ was calculated as follows:

4 $${\mathrm{VP}}_{(a)}=1-\frac{{\mathrm{Moi}}_{(v)}\cdot\rho_{(w)}}{{\mathrm{Moi}}_{(m)}\cdot\rho_{(w\&s)}}$$

where ${\rho }_{(w)}$ is the pore water density measured by the weighing method and ${\rho }_{(w\&s)}$ represents for the density of mixed sediment measured by the submerged method.

### Data collection on chronology and eutrophication history

Sediment dating data were updated by interpolation and extension based on the age-depth model established in [29, 34] (site C4), which was calculated by ^210^Pb_ex_ dating for sedimentation rate and ^137^Cs activity analysis for absolute age using the constant rate of supply (CRS) model [28, 35]. Coincidentally, the 1963 bomb test peak exactly labeled the depth of the 1962 damming event. In the subsequent analysis, we coded the establishment of the Chaohu Dam as a dummy variable (i.e., 1 or 0). Data on the past epilimnetic total phosphorus (TP) concentrations estimated by diatom-inferred TP (DI-TP) collected from [28] were used to quantify the long-term trend of the eutrophication degree of Chaohu Lake. The DI-TP was calculated using sedimentary fossil diatom assemblages with a DI-TP transfer function model developed from a set of 45 lakes in the middle and lower reaches of the Yangtze River [36]. Furthermore, available data on the percentage of diatoms estimated by sedimentary pigments (PDESP) from [34] were used for comparison and verification.

### Nucleic acid extraction, sequencing, and bioinformatic analysis

#### Nucleic acid extraction

DNA extraction from sediment samples was performed using the PowerSoil® DNA Isolation Kit (MoBio Laboratories Inc., Carlsbad, USA) following the manufacturer’s *Alternative PowerSoil Protocol for RNA and DNA from Low Biomass Soil*. The DNA quality was assessed by ratios of 260 nm/280 nm and 260 nm/230 nm using a NanoDrop spectrophotometer (ND-2000, Thermo Scientific, USA). The DNA extraction protocol applied to further amplicon and metagenomic sequencing.

#### 16S rRNA gene amplicon sequencing

PCR amplification was performed with the primer pair 515F (5′-GTGYCAGCMGCCGCGGTAA-3′) and 926R (5′-CCGYCAATTYMTTTRAGTTT-3′) targeting the SSU V4-V5 region, which targets both bacterial and archaeal domains [37, 38]. High-throughput sequencing was performed using the Illumina HiSeq2500 platform (2 × 250 paired ends, Illumina, San Diego, USA) at Biomarker Technologies Corporation, Beijing, China. We prepared two sample treatments before amplicon sequence processing: one kept individual samples separate (i.e., the 57 sediment subsamples), and the other combined seasonal samples by pooling raw sequencing data of temporal samples of the same depth, which consisted of eleven depth-related samples. The merged samples were set to identify general vertical patterns without seasonal variation (see Fig. 1 and Table S1). Details of PCR amplification and amplicon sequence processing are available in Text S2. Overall, the average effective sequence number was 73668 ± 619 (mean ± SD), CV = 0.84% for seasonal individual samples, and 390759 ± 56565, CV = 14% for seasonal merged samples. To minimize the bias of sequencing depth, each sample set’s OTU table was rarefied to the lowest number among samples as a standard number of sequences.

#### Metagenomic sequencing and assembly

We further performed metagenomic sequencing for the upper nine sediment layer samples (0 ~ 45 cm) to obtain a reliable functional profile. The quality and quantity of the extracted DNA were examined using a Qubit dsDNA HS Assay Kit on a Qubit 3.0 Fluorometer (Life Technologies, Carlsbad, CA, USA) and electrophoresis on a 1% agarose gel, respectively. Paired-end libraries (insert size, ~ 350 bp) were prepared using a VAHTS Universal Plus DNA Library Prep Kit for Illumina (Vazyme Biotech). The library was sequenced on an Illumina NovaSeq 6000 platform (Biomarker Technologies Co., Ltd., Beijing, China) using the PE150 mode. Adaptor removal and low-quality sequence filtering were conducted in Trimmomatic v0.33. In detail, reads with a quality score < 20 over a sliding window size of 50 bp or with a sequence length < 100 bp were filtered. The average number of clean reads we obtained was 21.3 M (~ 6.5 G clean data base) per sample (Table S2). The high-quality short reads were de novo assembled using MEGAHIT which makes use of succinct de Bruijn graphs [39]. Assembly quality was assessed using QUAST v2.3 [40]. Contigs with lengths greater than 300 bp were selected as the final assembly result for further gene annotation. The contig number, contig length statistics, and mapped ratio are listed in Table S3.

#### Gene annotation and functional prediction

The open reading frames (ORFs) from each assembled contig were predicted using MetaGeneMark v3.26 [41] with default parameters. The prediction statistics are available in Table S4. All predicted genes with a 95% sequence identity (90% coverage) were clustered using MMseqs2 [42] to remove sequence redundancy. Functional annotations of representative sequences of nonredundant gene catalogs were performed against the NCBI nonredundant protein database (NR) and the Kyoto Encyclopedia of Genes and Genomes database (KEGG) with an e-value cutoff of 1e^−5^ using DIAMOND [43]; a manually curated integrative database NCycDB was also used for metagenomic profiling of nitrogen cycling genes [44] (Table S5-S11). As complementary to shotgun metagenomics, the 16S rRNA gene sequences provided extra functional prediction via PICRUSt [45] and the FAPROTAX database (Functional Annotation of Prokaryotic Taxa) [46]; the former was used to estimate the total gene content relevant to S/N/CH_4_ metabolism based on available sequenced genomes, while the latter was used to estimate the potential of fermentation based on experimental evidence (Table S12).

### Community statistics

We performed principal coordinates analyses (PCoA) based on binary Jaccard (presence–absence) and Bray–Curtis (abundance-weighted) dissimilarities to depict both taxonomic and abundance-based variations in community composition. Permutational multivariate analysis of variance (PERMANOVA) was applied using the R package *vegan* to test the hypothesis that depth dominates the distribution of prokaryotes. The seasonal fluctuation of microbial community at each depth was measured as the multiple-timepoint Bray–Curtis dissimilarity using the function *beta.multi.abund* in the R package *betapart* [47, 48]. Unweighted pair-group method with arithmetic means (UPGMA) clustering based on Bray–Curtis distance was performed to identify sediment layering properties. The layering significance was tested by analysis of similarities (ANOSIM). To visualize the microbial community transition patterns, we conducted an OTU-sample bipartite network analysis in Cytoscape with an edge-weighted spring-embedded layout using a seasonally merged OTU abundance matrix. Before that, a base-10 logarithmic conversion was applied, and the OTUs with log10-transformed abundance less than 1 were filtered. To map the effect of environmental factors in energy metabolism, we computed Pearson’s correlations within environmental data and Mantel correlations between community features and environmental data based on merged samples using the R package *ggcor* [49]. We also performed detrended correspondence analysis (DCA) and redundancy analysis (RDA) in *vegan* to visualize and test the specific influences of each environmental factor on abundant microbial taxa.

### Random-forest identification of damming-sensitive taxa

We applied the random forest (RF) algorithm to help identify the damming-sensitive phyla (DSPs) and classes (DSCs), the key components that distinguish between the two sediment layers divided by the damming line mapped in the sediment profile. We first performed a supervised classification, i.e., given the classification strategy, the 57 sediment samples were divided into the pre-damming group and the post-damming group; 54 observed phyla (or 134 observed classes) were viewed as characteristic variables for classification. We used the mean decrease Gini (MDG) to evaluate variable importance. The higher the MDG is, the more critical the corresponding feature. Specifically, we constructed 100 RF replicates (each with 1000 trees for DSP or 2000 trees for DSC) using the R package *randomForest* [50] and pooled them using the function *combine* to obtain a robust importance ranking. Then, we computed the optimal breakpoints based on linear regression models via the function *breakpoints* implemented in the R package *strucchange* [51] to identify the DSP/DSCs that respond most strongly to the legacy effect. Classification accuracy was assessed using the out-of-bag (OOB) error rate. In addition, we also performed unsupervised RF clustering without prior classification information to verify the plausibility of the damming-line classification strategy.

### Quantifying microbial community assembly processes

To evaluate community assembly processes, we first performed variation partition analysis (VPA) using the function *varpart* in *vegan* [52]. This approach estimates the contribution of measured deterministic factors to the metacommunity assembly process and the importance of past events in altering the present environment. We classified all environmental variables into what we term “present parameters,” “sedimentary features,” and “historical parameters” (see details in Text S3). In each group, a stepwise model selection algorithm was applied to streamline the variable subset. These explanatory data frames were Hellinger transformed. We interpreted the unexplained fraction as stochastic composition.

Because there may be important unmeasured variables that influence community assembly, we used null model analysis based on phylogenetic information to infer underlying ecological processes. MNTD (mean nearest taxon distance) and βMNTD (i.e., between-assemblage analogs of MNTD) were calculated using *mntd* and *comdistnt* in the R package *picante* v1.8 [53]. Measuring standard deviations of observed MNTD/βMNTD from mean MNTD/βMNTD in the null model in which taxa are randomized (999 randomizations) across the tips of phylogenetic trees, NTI (nearest taxon index), and βNTI were calculated using *picante* and R code [54] to quantify the deviation from pure stochastic ecological processes governing local community structure and dynamics. The fraction of pairwise comparisons with significant βNTI values (|βNTI|> 2) indicates the influence of selection, while nonsignificant results (|βNTI|< 2) indicate stochastic processes [55]. To evaluate differences in the processes of community assembly across depths, we computed the pairwise comparisons of βNTI values between adjacent layers within each sediment column section. We then partitioned stochastic processes into dispersal limitation (i.e., low rates of dispersal leading to dissimilar community structure), homogenizing dispersal (i.e., high rates of dispersal leading to community homogenization), and ecological drift (i.e., undominated processes of birth, depth, and reproduction) by calculating the Bray–Curtis-based Raup–Crick metric (RC_bray_) by comparing empirically observed Bray–Curtis (BC_obs_) to simulated Bray–Curtis (BC_null_) under 9999 randomizations [54]. As such, the relative contributions of each ecological process in the assembly of communities under different categories (full system including all samples and the two-layer system clustered by UPGMA) were quantified based on the following rules: the fraction of all pairwise comparisons with |βNTI|> 2 was taken as an estimate for the influence of selection, |βNTI|< 2 and RC_bray_ > 0.95 as dispersal limitation, |βNTI|< 2 and RC_bray_ <  − 0.95 as homogenizing dispersal, and |βNTI|< 2 with |RC_bray_|< 0.95 as ecological drift (the undominated fraction). The selection process was further divided into homogeneous (βNΤI <  − 2) and heterogeneous (βNΤI > 2) types.

Once the metacommunity of the full system was proven deterministically assembled, the Levin’s niche theory [56] was applied to estimate each local community’s emergent niche property. Mean niche breadth $\overline{{B }_{i}}$ of a local community at a specific depth was calculated as follows:

5 $$\left\{\begin{array}{c}\overline{B_i}=\sum\nolimits_{j=1}^SQ_{ij}\cdot B_j\\B_j=1/\sum\nolimits_{i=1}^NP_{ij}^2\end{array}\right.$$

where ${B}_{j}$ is the Levin’s measure of the niche breadth of OTU $j$ across the metacommunity, ${Q}_{ij}$ represents the relative abundance of OTU $j$ in the local community $i$, ${P}_{ij}$ is the percentage of the OTU $j$ in environment $i$ to the total abundance of OTU $j$ across the metacommunity, $S$ is the total number of OTUs, and N is the total number of local communities.

### Interpreting emergent stochasticity via path modeling

We quantitively constructed the causality between historical damming and emergent stochasticity using partial least squares path modeling (PLS-PM) in the R package *plspm* [57]. Our modeling assumptions are (i) temporal environmental variations and spatial energy difference would be responsible for local community fluctuation and emergent stochasticity of community assemblage; (ii) historical damming and eutrophication would largely explain the spatial energy difference and strengthen the polarization of the oxic, nutrient-rich surface and the anoxic, barren deep sediments; (iii) compared to those with progressive changes, such layering property would narrow the metacommunity’s mean niche breadth (MNB) by shortening the spatial scale of community turnover, but the local MNB in the rapid transition zone would be larger where the generalists with high metabolic plasticity are selected; the larger the MNB, the more emergent stochasticity can be locally observed.

In measurement models, we set the legacy effect and environmental fluctuation as the two exogenous variables mutually independent. The latent variable “legacy effect” was constructed by Damming, $(1-PDESP)$, and DI-TP in a formative way; similarly, the variable “environmental fluctuation” was formed by coefficients of variation of seasonal moisture (${\mathrm{CV}}_{\mathrm{moi}}$) and temperature (${\mathrm{CV}}_{\mathrm{temp}}$) in each sediment layer (Fig. S11). Differently, “Energy difference” was set as an endogenous latent variable measured in a reflective way by chemotaxis and the difference between neighboring layers of both redox potential and TOC. “Community fluctuaton,” “mean niche breadth,” and “emergent stochasticity” were set as endogenous manifest variables measured by multiple-timepoint dissimilarity, MNB, and $(10-{\mathrm{\beta NTI}}_{\mathrm{merged}})$, respectively.

## Results

### Sediment dating, limnological information, and gaseous methane sequestration

**Fig. 2:**
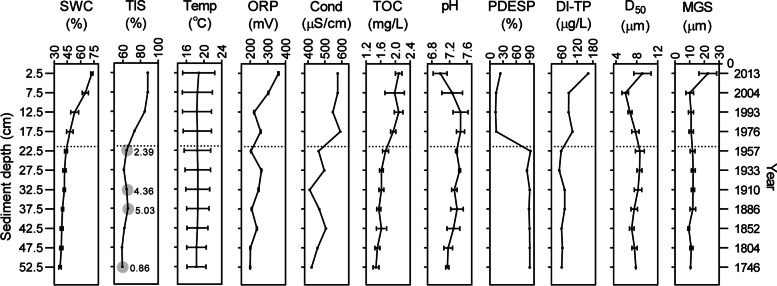
Vertical profiles of key environmental parameters in relation to sediment depth and age. Values with error bars are means (± 1 SEM) from six seasonal snapshots. SWC saturated (mass) water content (${\mathrm{Moi}}_{(m)}$), TIS total interstitial space volume percent, Temp sediment temperature, ORP redox potential compared to the standard hydrogen electrode (SHE), Cond conductivity, TOC total organic carbon, PDESP percentage of diatoms estimated by sedimentary pigments, DI-TP diatom-inferred total phosphorus, D_50_ particle median diameter, MGS mean grain size. Gray circles represent the existence of methane bubbles with gas volume (${\mathrm{VP}}_{(a)}$, %) labeled by the number aside. The dotted line labels the damming event

Sediment cores with the effective depth of 55 cm had a maximum age of ~ 306 years (Fig. 2 and Fig. S1). The age-depth model placed the ^137^Cs peak, a synchronous sign of the Chaohu Dam construction, at a depth of 21.4 cm within the fifth layer. The average deposition rate in the upper 21 cm (${\overline{r} }_{\mathrm{upper}}=0.41\mathrm{ cm}/\mathrm{year}$) rose over twofold compared to the pre-damming condition (${\overline{r} }_{\mathrm{lower}}=0.13\mathrm{ cm}/\mathrm{year}$). Diatom stratigraphic data showed a marked transition since damming with a succession of diatom-cyanobacteria in 1966 [34] and elevated nutrient loading in the 1970s [28]. Accordingly, we termed the 21.4-cm-depth (or the fifth layer) the damming-labeled horizon (DH), and the fourth and fifth layers the historical transition zone (HTZ) that included both damming and eutrophication, hereinafter. With redox potential decreasing rapidly in the first two layers, the sediment color changed from brown to charcoal gray, indicating a Fe^3+^/Fe^2+^ transition in the top 10 cm. Sediment texture also shifted between the first two layers, from the silt loam of the top layer (${\overline{{\mathrm{MGS}}} }_{L1}$ = 22.38 μm) to the deeper silty clay loam (${\overline{{\mathrm{MGS}}} }_{L2\sim L11}$ = 11.20 ± 1.02 μm). Decoupled with the sediment grain size, sediment total interstitial space volume (TIS) dropped in HTZ and stabilized at approximately 60% in deep. Interestingly, bubble formation hinting methane oversaturation occurred only below DH that contributed to the “local TIS”. This patten indicated that dam building has the potential to affect greenhouse gas emissions by influencing the depth of gaseous methane sequestration in sediment.

### Vertical organization of sediment microbial community

**Fig. 3:**
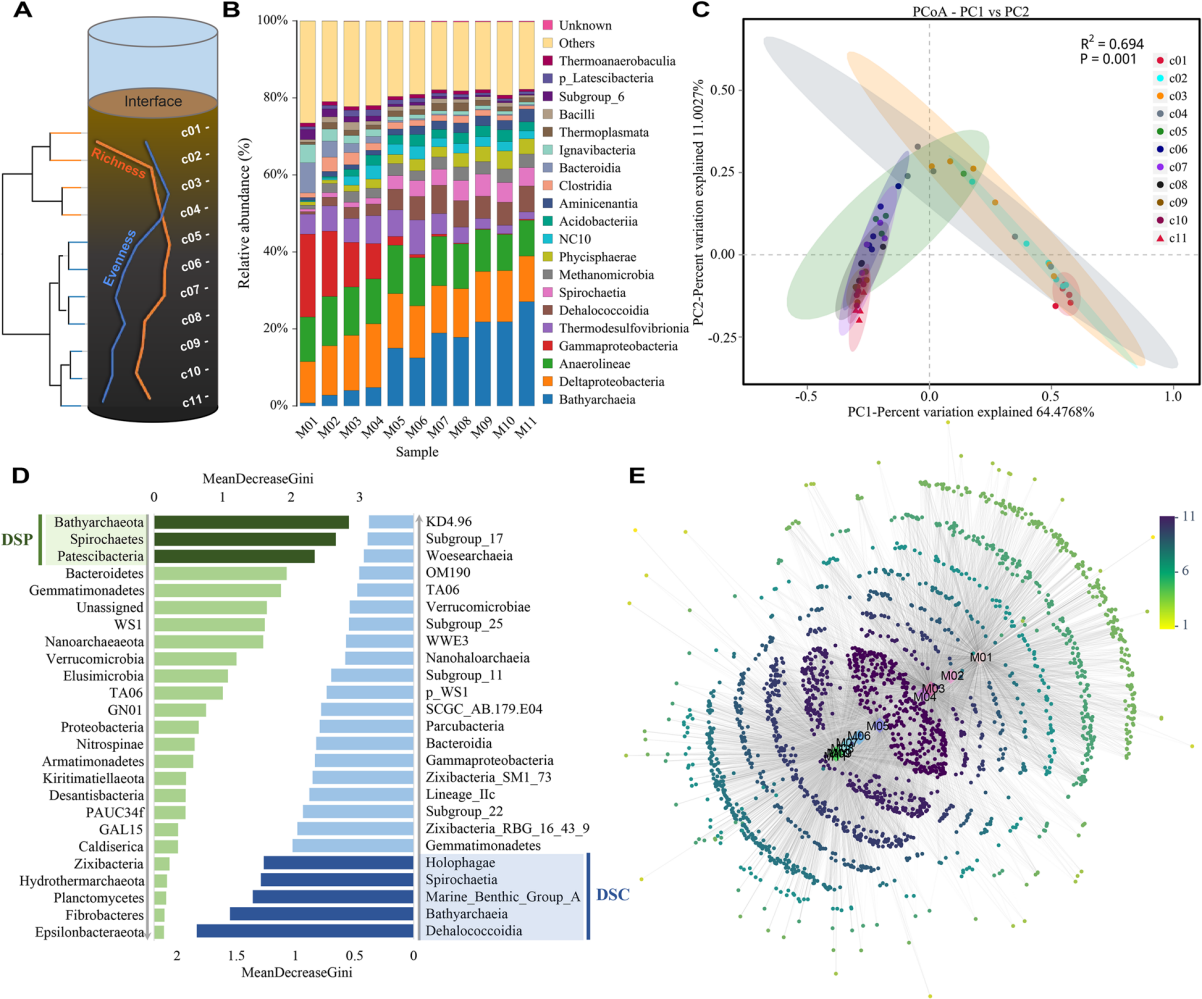
Vertical organization of sediment microbial community. **A** Overview on sediment layering structure, including alpha diversity shifts along sediment depth and clustering analysis. Orange line stands for the richness of Chao1 ($S\in <span class='reftype'><span class='reftype'><span class='reftype'>[1745, 2180]</span></span></span>$) and blue line for Pielou’s evenness ($E\in [0.76, 0.86]$). UPGMA divided eleven spatial samples into two main clusters (*R* = 0.974, *P* = 0.002): the upper four layers and the lower seven layers, coinciding with the historical damming event. **B** Composition of dominant classes (the top 20) in different depth layers. **C** Principal coordinates analysis (PCoA) based on Bray–Curtis dissimilarities of OTUs illustrating continuous changes among bacterial communities grouped by depth. The size of 95% confidence circles shows within-group dissimilarities that represent seasonal fluctuation. **D** Identification of damming-sensitive phyla (DSP) and classes (DSC) in sediment based on random forest classification. The mean decrease in Gini value identifies the most reliable and relevant predictors (phyla/classes) to perform classifications. **E** Emergent stratification via bipartite network analysis with eleven seasonally merged samples. The node color shifting from yellow toward dark purple indicates a higher OTU-sample connection degree counting from 1 to 11. The grayscale of lines is indicative of the OTU richness in one sample

The sediment profiles displayed a well-organized structure wherein dam construction made a sharp transition nested within a progressive turnover (Fig. 3). Species abundance difference rather than taxonomic turnover accounted for the high beta diversity (Fig. S2). The discrete stratification was quantitatively validated by UPGMA (Fig. 3A) and unsupervised RF clustering (Fig. S5). The PCoA plot (Fig. 3C) further depicted the distinct shift, while the size of the 95% confidence ellipse, representing seasonal fluctuation, exhibited a spindle-shaped distribution patten with a peak at the fourth layer. This indicated a seasonally variable community within the sharp transition zone. Notably, the OTU particles in the topological network (Fig. 3E) displayed an “interference” pattern resembling a “two-wave superposition,” suggesting the segregation of the metacommunity into opposing factions. This polarization corresponded to the stratification at DH, signifying the contrast between the oxygen-rich, nutrient-abundant surface and the anoxic, nutrient-poor deep sediments. Moreover, the local communities surrounding DH exhibited higher species richness (Fig. 3A) and a greater overlap of species (i.e., higher connection degree, Fig. 3E), indicating an ecotonal environment.

As the most abundant taxa, *Gammaproteobacteria* and *Bathyarchaeia* were the representative classes of the surface and deep communities, respectively (Fig. 3B and Fig. S6). We further identified the damming-sensitive taxa that displayed an evident shift at the depth labeled by the damming event (Fig. 3D). The supervised RF classifier identified *Bathyarchaeota*, *Spirochaetes*, and *Patescibacteria* as the damming-sensitive phyla (DSPs) and identified *Dehalococcoidia*, *Bathyarchaeia*, Marine Benthic Group A (MBG-A), *Spirochaetia*, and *Holophagae* as the damming-sensitive classes (DSCs) with good classification accuracy (see Fig. S3 and S4). Except for *Holophagae* (*Acidobacteria*), the rest of the DSPs/DSCs were all deep adaptors that exhibited a sharp (bottom-up) decline at DH.

### Biogeochemical zones, energy difference, and community stability

**Fig. 4:**
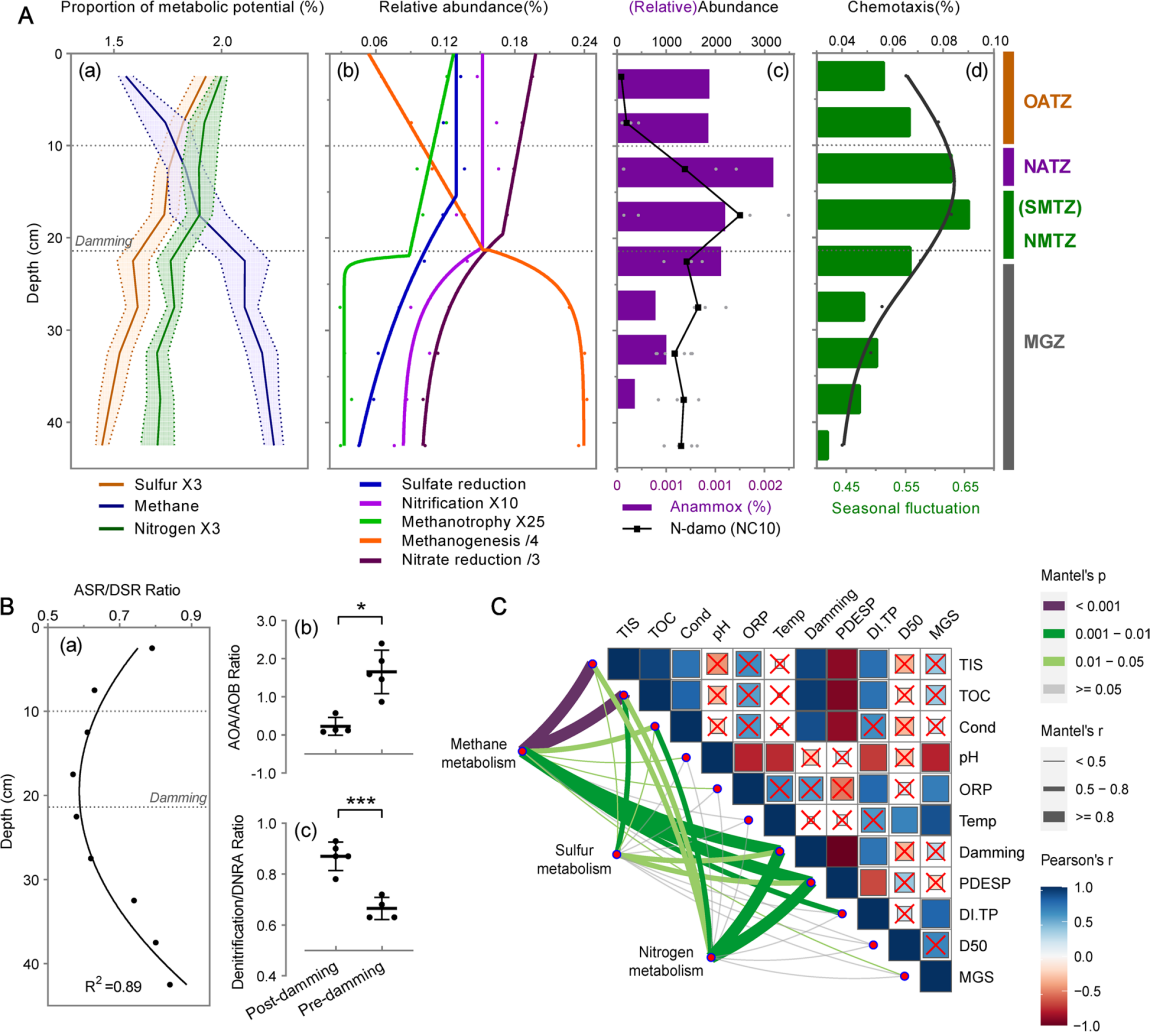
Vertical biogeochemical zonation according to patterns of energy metabolism. Total S/N/CH_4_ metabolic abundances with seasonal variations (**A**a) were predicted with 16S rRNA genes; specific function modules and chemotaxis (**A**b–d) were predicted by metagenomic data annotated with KEGG and NCycDB. Sulfate reduction (*R*^2^ = 0.975, *df* = 5) and nitrification (*R*^2^ = 0.854, *df* = 6) were fitted with exponential model of plateau followed by one phase decay. Methanotrophy (*R*^2^ = 0.983, *df* = 4), methanogenesis (*R*^2^ = 0.999, *df* = 4), and nitrate reduction (*R*^2^ = 0.998, *df* = 4) were fitted piecewise with a linear model followed by exponential one-phase decay/association. Least squares fit was used for all these nonlinear models. Relative abundance of the NC10 class (**A**c) serves as proxy for the intensity of N-damo, the nitrate/nitrite-dependent anaerobic methane oxidation. Black dots in plot **A**c represent the median value of the seasonal samples labeled by gray dots. OATZ oxic–anoxic transition zone, NATZ nitrate–ammonium transition zone, SMTZ sulfate–methane transition zone, NMTZ nitrate/nitrite–methane transition zone, MGZ methanogenetic zone. **B** Several ratio indexes relevant to S/N-cycling metabolism. ASR/DSR ratio of assimilatory/dissimilatory sulfate reduction (quadratic fitting). AOA/AOB ratio of ammonia-oxidizing archaea and bacteria reflected by archaeal/bacterial amoABC genes (Mann–Whitney test with *P* = 0.016*, normality test failed), Denitrification/DNRA ratio of denitrification and dissimilatory nitrate reduction to ammonium (unpaired *t* test with *P* = 0.0003***, Shapiro–Wilk normality test passed). **C** Environmental drivers of energy metabolism. Line color represents Mantel’s *p* and line width represents Mantel’s *r*. Red “X” means no significance for Pearson’s correlation

We characterized GTZs based on the profile of microbial metabolic potential predicted by metagenomic and 16S rRNA gene sequencing (Fig. 4A and Table S5-S10). To characterize the process of anaerobic methane oxidation coupled to denitrification (N-damo), the typical N-damo bacteria phylum NC10 was selected as an indicator [58–61]. Generally, the relative abundance of sulfur and nitrogen metabolic genes shared a similar fading tendency with two rapid drops in the top 10 cm and around the DH, while methane metabolism had a reverse trend. We then defined GTZs accordingly: the top 10 cm of sediment was defined as an OATZ in which redox potential monotonically decreased (see ORP in Fig. 2) and the relative abundance of N-damo approached zero; the 10 ~ 15 cmblf (cm below lake floor) right below the OATZ was characterized as a NATZ where a hotspot for ongoing anaerobic ammonium oxidation (anammox) was observed, indicating a transition of dominant N species from nitrate to ammonium [3]; sediment below DH was defined as a methanogenetic zone (MGZ) where methanotrophs decreased whereas fermentation and methanogenesis quickly increased (Fig. 4Ab, and Fig. S7). It was consistent with the pattern of gaseous CH_4_ accumulation in Fig. 2. In HTZ (15 ~ 25 cmblf), both sulfate and nitrite/nitrate reduction potentials dropped rapidly, the former occurred at approximately 15 cmblf while the latter at the DH. The replacement between methanogenesis and nitrate/nitrite reduction at the DH indicated the system’s preference for NMTZ rather than SMTZ. Active AOM coupled to denitrification was confirmed by the pronounced peak of N-damo right above the DH. Interestingly, the ratio of assimilatory/dissimilatory sulfate reduction (ASR/DSR) exhibited a quadratic distribution with minimum value in HTZ (Fig. 4Ba and Table S8), supporting the occurrence of AOM coupled to (dissimilatory) sulfate reduction because the DSR’s efficiency in accepting electrons is much higher than that of ASR [62]. In addition, the ratio of ammonia-oxidizing archaea and bacteria (AOA/AOB) and the ratio of denitrification and dissimilatory nitrate reduction to ammonium (DEN/DNRA) also showed significant differences at DH (Fig. 4Bb-c).

**Fig. 5:**
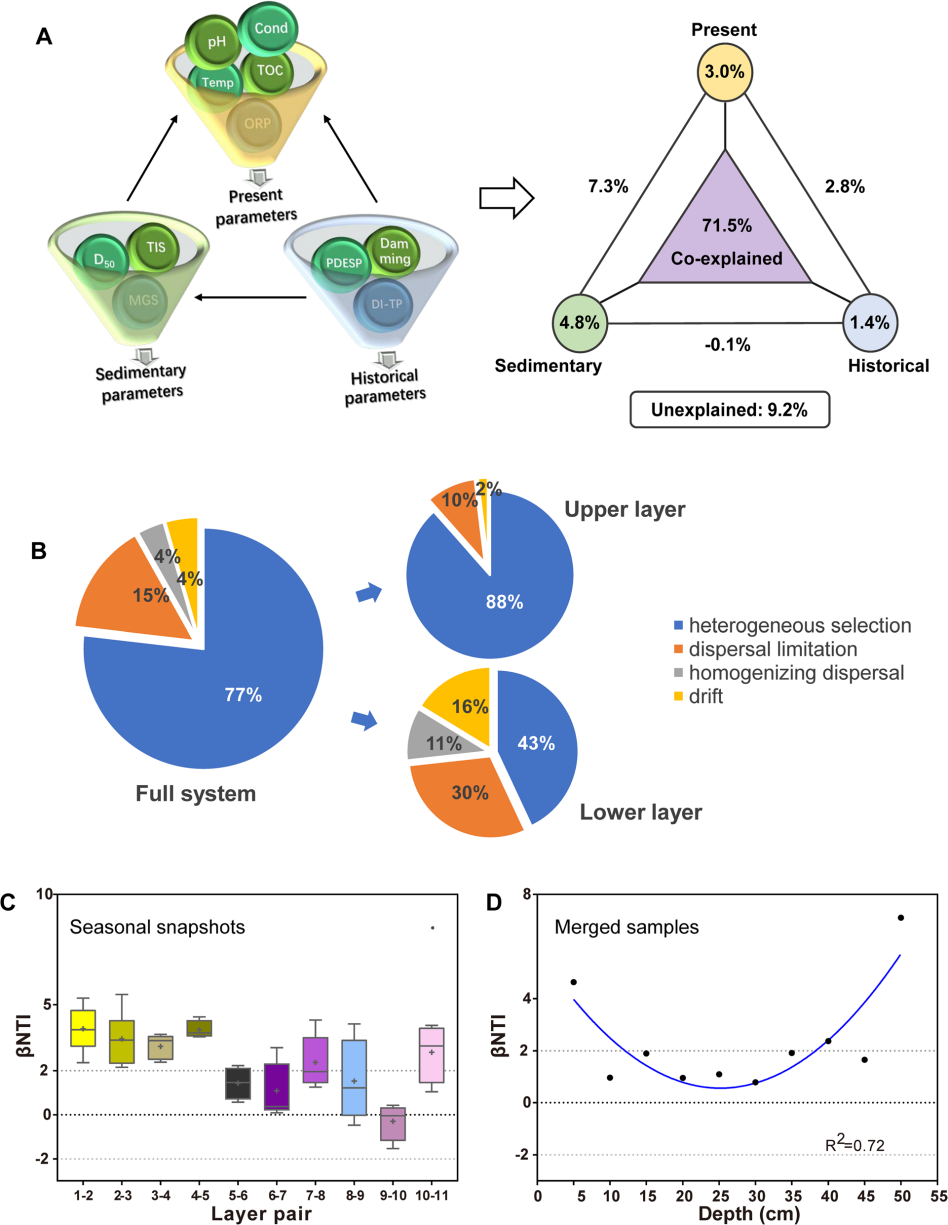
Quantitative estimates of the microbial community assembly process across sediment depths.** A** Variance partition analysis (VPA) revealing the relative contributions of contemporary, historical, and sedimentary parameters to the whole sediment community variations (Hellinger transformed). **B** Different ecological processes in assembly of microbial communities before and after damming. **C** Different patterns of βNTI between neighboring layers across sediment using the data of individual samples and seasonally merged samples. A quadratic model was applied for eleven merged samples. The upper and lower whiskers of each box correspondingly denote the minimum and maximum values. Median, mean values, and outliers are shown by the midline, plus dot, and black dot, respectively. The area between βNTI =  + 2 and − 2 denotes stochastic assembly

Obviously, historical damming heavily influenced the vertical biogeochemical zonation via affecting microbial energy metabolism, especially the methane and nitrogen metabolism (Fig. 4C). High correlations between historical (PDESP/DI-TP/Damming) and present parameters (TIS/TOC/Cond) reflected that damming and eutrophication have caused permanent changes in current sediment physicochemical traits (Fig. 4C and Fig. 5A). Throughout the sediment system, TIS, TOC, and Cond rather than ORP became the three most relevant contemporary environmental variables on which strong legacy effects were shed by damming and eutrophication.

Spatiotemporal heterogeneity of energy supply and prokaryotic active dispersal ability were further reflected by chemotaxis (Fig. 4Ad, Text S4, and Table S11). Metagenomic data showed that the relative abundance of chemotaxis genes peaked in NATZ and NMTZ, and remained low below DH, indicating a large energy tension within HTZ and a more homogeneous, infertile environment below DH. This chemotaxis pattern also reflected microbial flexibility in environmental adaptation. Interestingly, the pattern of seasonal community fluctuation, also illustrated by the size of the 95% confidence ellipse in the PCoA plot (Fig. 3C), was in sync with the pattern of chemotaxis, indicating a more variable local community in HTZ over time. Both the multi-timepoint dissimilarity and microbial chemotaxis served as indicators of community stability.

### Deterministic processes govern metacommunity dynamics

Variation partition analysis (VPA) showed that the measured contemporary, historical, and sedimentary variables jointly explained the basic microbial community variations (explained variation = 90.8%, Fig. 5A), indicating a deterministic nature of community assembly processes and most of the drivers had been found. The high co-explained variance percentage (71.5%) implied the historical context dependency of the current environment. The null model further validated the determinism as inferred from VPA. For the full system, heterogeneous selection dominated the assembly of the sediment metacommunity and dispersal limitation came next at 15% (Fig. 5B and Fig. S9A). Communities of seasonal snapshots and merged samples were all significantly phylogenetically locally clustered, which provided strong evidence of environmental filtering (NTI > 2, Fig. S8). In addition, a positive relationship formed in the plot of pairwise phylo-betadiversity versus spatial distance (Fig. S9B), indicating a distance decay of phylogenetic similarity. Given the depth dependence of environmental variables, environmental selection acts as an overwhelming factor on phylogenetic turnover rather than geographical isolation [63].

**Fig. 6:**
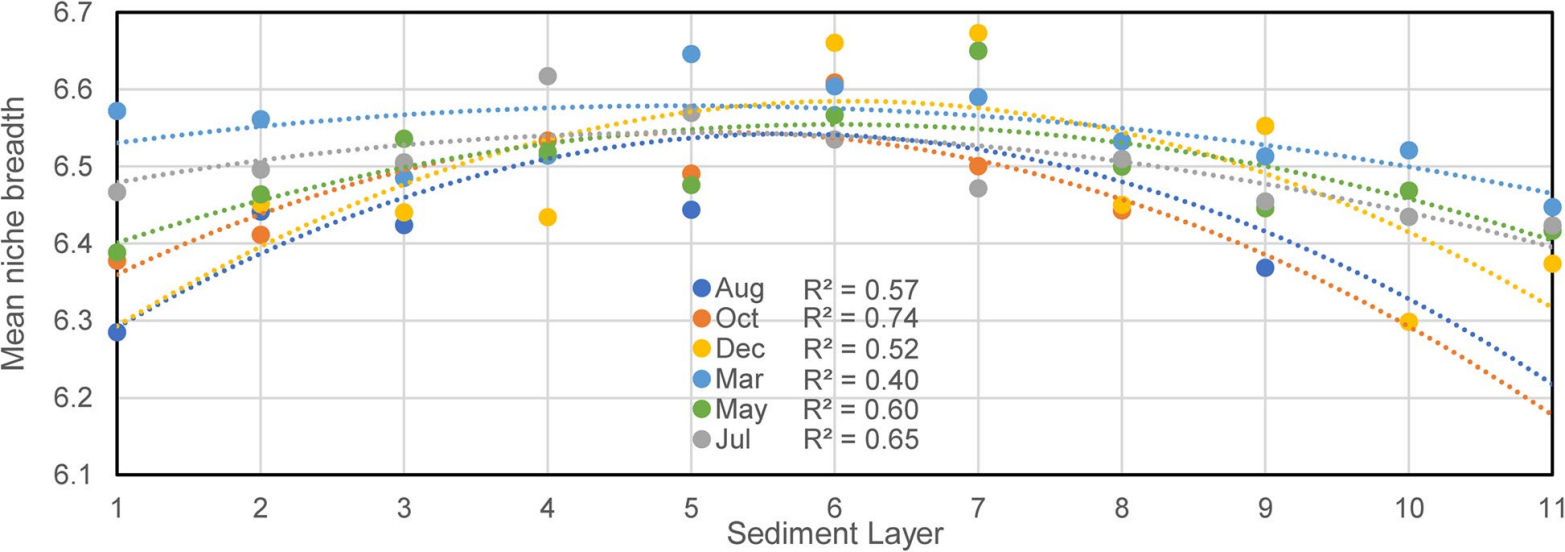
Patterns of mean Levin’s niche breadth index of local communities across depths in different months fitted by second-order polynomial model based on ordinary least squares regression. The unit of MNB is layer(s) ($MNB\in <span class='reftype'><span class='reftype'><span class='reftype'>[0, 11]</span></span></span>$). A higher MNB value indicates that the community harbors more generalist taxa that are more uniformly distributed across environments, while low value indicates that the community harbors more specialists that favor specific environments

In the context of niche construction, we observed a robust parabolic pattern of MNB across depth (Fig. 6 and Fig. S10). The peak position showed that communities at intermediate depth harbored more generalists with higher metabolic flexibility, while those close to the two relative extremes harbored more specialists. It corresponded to the OTU connection degree pattern in the bipartite network (Fig. 3E), hinting at a more inclusive ecotonal environment where the fertile upper layers and the low-energy deep sediments met.

### Assembly stratification and temporal emergent stochasticity at the damming horizon

**Fig. 7:**
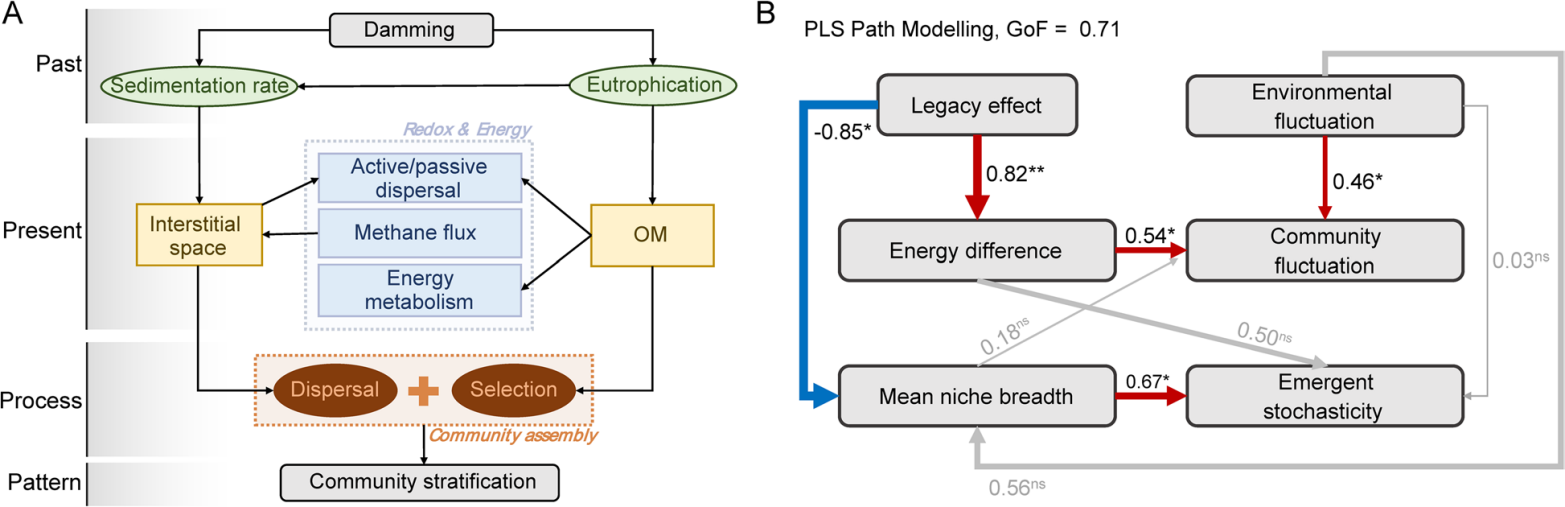
**A** Hypothesized mechanism of microbial community stratification. The damming in 1963 accelerated lake eutrophication and sedimentation rate, altering sediment nutrient composition and increasing energy input. This changed the metabolic type, order, intensity, and chemotaxis of sediment microorganisms, shortening the natural sediment profile transition process. On the other hand, accelerated sedimentation rate, enhanced sediment biodegradation rate, and gaseous metabolites jointly imposed the interstitial space, which further affected the negative dispersal of sediment microbes. As a result, the relative proportion of dispersal and selection shifted and resulted in community stratification. OM organic matters. **B** Partial least squares path modeling (PLS-PM) quantitatively depicting the cause-effect mechanisms underlying the emergent stochasticity at the sharp transition zone revealed by the time-merged data. Red and blue arrows represent significant (* *P* < 0.05 and ** *P* < 0.01) positive and negative paths, respectively; paths with no significance (ns, *P* > 0.05) are labeled in gray. The arrow width reflects the path coefficient value (the number near the path). The goodness-of-fit (GoF) shows a good prediction power of the model that is of 71%. Specific assessment indexes of both measurements (Cronbach’s alpha, loadings, etc.) and structural models (*R*^2^, redundancy, etc.) are listed in Table S13

We performed pairwise comparisons between adjacent layers to show differences in the process of community assembly across depth. Intriguingly, two sample treatments (snapshots vs. seasonally merged) exhibited distinct βNTI patterns (Fig. 5C). The snapshot (individual) sample set displayed a pronounced shift at DH, from heterogeneous-selection-oriented deterministic community assembly (βNTI > 2) to a more stochastic one (|βNTI|< 2 except the bottom layer). Dispersal limitation accounted for a relatively high proportion especially below DH where it tripled in proportion (Fig. 5B). We sketched a potential mechanism by which GTZs overlap with historical damming is not a coincidence: damming has profoundly changed the assembly processes of sediment microbial communities by enhancing selection and reducing dispersal limitation (Fig. 7A).

In contrast, adjacent seasonally merged sample pairs exhibited a distinct pattern of U-quadratic distribution pattern (*R*^2^ = 0.717, Fig. 5C) in which the two opposite extremes showed significant deterministic processes, while the local communities around the rapid transition were more stochastically assembled. The over-time emergent stochasticity potentially echoed the high OTU connection degree and MNB at DH, as well as the high chemotaxis and community fluctuation in HTZ. PLS-PM revealed their causal relationships (Fig. 7B and Table S13). First, the energy difference pattern (reflected by chemotaxis, *Δ*_TOC_, and *Δ*_ORP_) was largely contributed by historical damming. Second, the spatial energy difference and seasonal environmental fluctuation (formed by ${\mathrm{CV}}_{\mathrm{moi}}$ and ${\mathrm{CV}}_{\mathrm{temp}}$, Fig. S11) jointly accounted for the community fluctuation pattern over time; comparatively, the energy difference had more explanatory power. Third, the negative correlation between the legacy effect and MNB as well as the positive correlation between MNB and emergent stochasticity well confirmed our hypotheses that the sediment polarization induced by damming would shorten the regional MNB but widen the local MNB at DH, which explained the emergent stochasticity there (see also in the section “Methods”).

## Discussion

Although there is a long-standing interest in sediment zonation, anthropogenic activity is a critical yet widely overlooked determinant of sediment structure in freshwater lakes. Here we provide multifaceted evidence that historical dam construction dramatically influences sediment microbial stratification in taxonomic diversity, energy metabolism, and community assembly processes. As a result, the redox hierarchy of GTZs is altered with NMTZ controlling the burial depth of gaseous methane. Moreover, temporal investigations enable us to observe the emergent stochasticity around the damming horizon, behind which lurks the impact of stratification on community stability and metabolic flexibility. Our findings provide a novel perspective on the formation mechanism and ecological properties of sediment stratification in freshwater systems.

### Formation mechanism of stratification at DH: metabolic differentiation

Primarily, historical damming triggers the initial differentiation at DH. The initial differentiation lies in the matter/energy input and the geophysical properties. Dam construction can significantly enhance sedimentation and trap nutrients in reservoirs by increasing hydraulic residence time and reducing flow velocity [22]. In Chaohu Lake, dam construction brought a twofold increase in sedimentation rate and induced eutrophication in that TN/TP rapidly increased and the organic carbon burial rate doubled [64]. Meanwhile, dam operations modify substrate characteristics [65]. We observed a rise in sediment porosity after damming but no obvious change in grain size (Fig. 2), indicating the effect of the elevated sedimentation rate on the looseness of the stacked structure rather than on particle size. This may be one of the geophysical points that distinguish natural burial processes and those with exogenous contingency (damming). Notably, the differentiation was nested in the process of sediment aging that never broke the continuity of the natural, slow-rolling transition of oxygen, energy, and porosity.

While enhanced burial rate and lake trophic level widen the gap of microbial nutrient availability, the initial difference is further amplified by microbial metabolic activities (Fig. 7A). Below DH, relatively low initial nutrient concentration coupled with natural energy depletion with burial results in the exhaustion of available electron acceptors. It weakens anaerobic methane oxidation and enhances fermentation (Fig. 4A and Fig. S7). Fermenters produce methanogenic precursors (e.g., hydrogen and acetate) and push forward the reaction of methanogenesis which lacks consumers, thus accelerating methane accumulation, leading to methane boiling and upward diffusion. When the gas reaches up to DH where available external electron acceptors (e.g., nitrate/nitrite and sulfate) are ample, anaerobic methane oxidation coupled with denitrification and dissimilatory sulfate reduction rapidly consumes methane, forming a convergence barrier and intensifying metabolic niche differentiation. This damming-adjusted metabolic cascade facilitates methane sequestration (Fig. 2). Although the gaseous CH_4_ emission magnitude of dammed, eutrophic reservoirs has been reported an order larger than that of natural oligotrophic reservoirs [66], how such a sediment methane sequestration mode would affect the greenhouse gas emission progress (delayed release or permanent sequestrated) deserves further investigation.

Intriguingly, disobeying the order of free energy yield in the *Redox Tower*, the niche of N-dependent methanotrophs penetrated deeper than that of S-dependent methanotrophs. This can be explained by N excess: the metabolic potential of N was much higher than that of S across the observed profile; anthropogenic N input and accelerated sedimentation rate after damming further intensified the tendency. When sufficient nitrates coexisted with sulfate, they would be consumed preferentially as more efficient electron acceptors; consequently, the upward methane flux was controlled by the downward diffusion of nitrate/nitrite instead of sulfate. Analogous to the prevalence of SMTZ in S-dominated marine systems, N-AOM and NMTZ may prevail in both waterbody [5, 67] and sediment [4, 68] profiles of N-dominated freshwater systems. However, since the first discovery of N-damo in 2006 [59] and the evidence of N-damo as the major methane sink in stable freshwater environments in 2014 [4, 69], the concept “NMTZ” has not been well established in freshwater sediments; this study is among the first few ones to add NMTZ into the GTZ system of freshwater sediments (especially those in the eutrophic state).

Regarding dissimilatory nitrate reduction processes, the niche segregation of denitrifiers and DNRA bacteria is an important metabolic differentiation at DH (Fig. 4Bc and Table S5 ). The competition for nitrate between DNRA and denitrification has always been a hot issue. Our observation supports the mainstream view that it is closely related to the ratio of available electron donor (i.e., degradable carbon) and electron acceptor (i.e., nitrate) [70, 71] as well as the O_2_ status [72, 73]. The proportion of denitrification (nitrate → nitrogen) above DH is significantly higher because microaerophilic denitrifiers are favored under high nitrate (low C/NO_3_^−^ ratio) and low-O_2_-supply conditions; anaerobic DNRA (nitrate → ammonia) predominates below DH because DNRA is favored by nitrate attenuation (high C/NO_3_^−^ ratio) and a rigorously anoxic environment. From the view of electron-transport efficiency, three more electrons are transferred during DNRA (NO_3_^−^  + 10H^+^  + 8e^−^  → NH_4_^+^  + 3H_2_O) than denitrification (2NO_3_^−^  + 12H^+^  + 10e^−^  → N_2_ + 6H_2_O) so that a more reductive condition with high ratio of electron donor to acceptor is more prone to favor DNRA [70, 73]. In fact, it is also a natural selection of N equilibrium: denitrification relieves N excess above DH by converting NO_3_^−^ to gaseous N_2_, while DNRA retains N below DH by converting easily eluviated nitrate–anions into easily adsorbed ammonium ions. Interestingly, this strategy converges the difference in N amount between the upper and lower layers but enhances the difference in redox potential. Below DH, excessive carbon substrates would activate fermentative bacteria [74, 75]; fermentative DNRA yields a large number of reduction products that make the environment more reductive; strong reductive conditions in turn benefit to DNRA, and one of the products, acetate (nonfermentable substrate), can be further utilized as an electron donor by respiratory DNRA [76].

### Formation mechanism of stratification at DH: taxonomic differentiation

Metabolic differentiation is further reflected by microbial taxonomy in diverse ways. The most intuitive is the niche differentiation of bacteria and archaea (see the bacteria–archaea proportion in Table S4, the representative classes and DSCs [*Bathyachaeia* and MBG-A] in Fig. 3B and D, and the AOA/AOB ratio in Fig. 4Bb). With deep-branching phylogeny and specific niche adaptation to deep, anoxic subsurface environments, it is not surprising that archaea exhibit such a sudden decline [15]. For the structuring lineage *Bathyarchaeota* (formerly known as the Miscellaneous Crenarchaeotal Group, MCG), the capabilities of encoding *mcr* genes [77] and utilizing recalcitrant organic matter [78, 79] are their representative metabolic characteristics to inhabit below DH where methanogenesis and slow degradation of refractory organics predominate. Likewise, the *No.1* DSC *Dehalococcoidia* (*Chloroflexi*) prefer deep sediments for their metabolic characteristic of rigorously anaerobic organohalide respiration. Their niche preference for deep environment has also been reported in Lake Stechlin [15], Lake Baikal [80], deep sediments of ridge flank environments [81], and bottom waters of an Arctic lake [82].

Legacy effect on the *No.3* DSP *Patescibacteria* results from another metabolic strategy: to be a minimalist. They have ultrasmall cell sizes and ultrasimplified genomes (~ 1 Mbp). By streamlining nonessential functions such as flagellar assembly and stress response systems, they do not invest in motility and chemotaxis, and thus cannot adapt well to the upper sediment environment with high heterogeneity and perturbation, but adapt well to nutrient-limiting deep sediments by effectively reducing energy consumption [83]. In this context, the phylum *Patescibacteria* is on behalf of those who contribute to the low chemotaxis pattern below DH (Fig. 5). In contrast, the *No.2* DSP *Spirochaetes* are motile, but in a unique way via the helical shape of their cells and the polar positioning of their axial filaments, which help them easily move through deep clay sediments where the high viscosity and small TIS block the movement of most flagellated microbes [84]. Chemotactic responses supported by such a unique motile strategy make *Spirochaetes* more competitive in nutrient-poor environments. Besides, they generate ATP during starvation by metabolizing endogenous RNA to survive in environments with extremely low energy [85].

Overall, these damming-sensitive taxa potentially indicate the ecological consequences of dam construction. Their unique adaptive strategies provide insights into community polarization and the alterations induced by damming, including changes in sediment porosity, nutrient availability, and redox condition. Their presence also supports the analogy between sediment stratification and an ecotone, where species transitions play a distinguishing role.

### Formation mechanism of stratification at DH: community assembly processes

Consistent with studies in other subsurface systems [55, 86, 87], deterministic processes dominated the microbial community assembly at the full-sediment-system scale. Specifically, individual samples showed that sediments buried after damming exhibited deterministic assembly, whereas the below-DH system was more stochasticly assembled where dispersal limitation governs ~ 30% turnover (Fig. 5B and C). On the one hand, dam construction and subsequent eutrophication enhance energy inputs and environmental heterogeneity; sufficient energy fuels microorganisms while sophisticated element cycling and complex substrate composition drive them to move actively (i.e., enhancing chemotaxis, Fig. 4 Ad). In contrast, sediments below DH harbor a relatively stable and barren deep biosphere where cell maintenance and survival (e.g., dormancy) predominates over cell synthesis, reproduction, and motility [15, 88–90], accounting for the high proportion of dispersal limitation.

On the other hand, sediment porosity has been considered another key driver of bacterial community assembly in hyporheic zones [54, 86, 91]. Highly permeable sediments (~ 4% mud) are considered associated with high levels of homogenizing dispersal, while fine-grained (~ 90% mud) texture restricts vertical water exchange and imposes dispersal limitation and selection [54]. Our sediment system can be categorized as the fine-grained group (Fig. 2); however, the accelerated burial rate after damming significantly enlarged the sediment interstitial space which provided channels to promote both active and passive dispersal of microorganisms. Comparatively, the lower TIS below DH probably accounts for the dispersal limitation and thus the stochasticity there. In brief, spatial variations of sediment interstitial space and nutrient induced by anthropogenic activities co-explain the layering pattern of community assembly processes, which in turn permanently impose the community stratification at DH (Fig. 7A).

### Sediment zonation acting as an ecotone: community stability, metabolic flexibility, and emergent stochasticity

The concept of an “ecotone”, traditionally applied to macroecological systems, finds relevance in describing sediment microbial stratification. The sharp transition observed in sediment microbial metacommunities exhibits several characteristics of an ecotone [23]: (i) a distinct line marked by damming, indicating a relatively abrupt change, (ii) shifts in microbial metabolism as key indicators, (iii) shifts of species, represented by damming-sensitive taxa, as a signal of differentiation, (iv) the “ecotone effect” manifested by increased richness compared to neighboring habitats, and (v) high metabolic flexibility and temporal heterogeneity within the ecotone, corresponding to the patterns of chemotaxis, mean niche breadth, and seasonal fluctuation. Thus, while it is not a term typically used to describe microbial communities, it can still be applicable in describing sediment microbial stratification. By exploring microbial ecotones, we broaden our understanding beyond macro-scale systems.

It seems a general rule in macroecology that ecotonal communities are unstable and easily predisposed to observational scaling effects due to spatiotemporal variability [23]. Our results well support this viewpoint that two sample treatments (snapshots vs. seasonally merged) exhibit distinct community assembly patterns along the sediment profile which echoes the community stability pattern. It has been proven from the perspective of complex system dynamics that high richness and strong species interactions directly contribute to persistent community oscillation [92–94] and in turn this oscillation maintains species diversity [95]. Here we observed higher species richness (Fig. 3A) and more coupled metabolism and co-metabolism at DH (Fig. 4), which indicated enhanced species interactions. In this context, the emergent stochasticity and instability at DH probably derive from the ecological network’s intrinsic property rather than environmental stochastic fluctuation.

On the other hand, dam construction has formed a new energy hotspot providing larger energy tension and acting as an attractor that greatly improves microbial metabolic flexibility by enhancing cell chemotaxis and recruiting more habitat generalists in HTZ (i.e., high MNB). High metabolic plasticity contributes to emergent stochasticity probably via ecological or/and statistical neutralities. It may directly enhance individuals’ neutral processes such as random dispersal, as microbes invest more in motility with the increase of energy and environmental heterogeneity. Emergent stochasticity may also come from the macroscopic perception of numerous independent deterministic events that are coarse-grained when scaling up, which brings statistical neutrality. Seasonal directional migration is one of these cases. A previous study reported that magnetotactic bacteria can migrate vertically in response to temperature fluctuations in lake sediments [96]. Microbes might migrate to warmer deep sediments in winter and to surfaces in summer, which is likely a universal yet overlooked hibernation strategy for those motile non-sporulating prokaryotes. Particularly, anthropogenic damming facilitates this strategy at the rapid transition zone by increasing energy tension and providing a low-energy, less competitive but warmer environment for microbial hibernation, thus facilitating the emergent stochasticity over time. Relevant contents remain to be further verified on finer spatiotemporal scales. In short, high community flexibility potentially leads to both ecological and statistical neutralities, resulting in the emergence of stochasticity.

## Conclusions

In-depth exploration of the anthropogenic pressure on the freshwater sedimentary biosphere is critical for better understanding the general mechanism of sediment biosphere formation. This study illustrates a unique sediment stratification pattern where historical dam construction significantly changes the redox order and microbial community structure, stability, and assembly process. We also emphasize that the nitrate–methane transition can be strengthened by such historical damming events, which play an important role in controlling methane sequestration depth. Although further studies are required to extend these findings in other aquatic sediments, it is probable that anthropogenic modification is a universal key factor shaping sediment microbial zonation and altering community assembly processes since sophisticated natural selection will not neglect any subtle change.

### Supplementary Information

**Additional file 1:** **Text S1.** Introduction to the Chaohu Lake and damming history. **Text S2.** PCR amplification and 16S amplicon sequence processing. **Text S3.** Classification of environmental factors. **Text S4.** Prokaryotic motility and chemotaxis. **Table S1. **Sample summary and naming scheme. **Table S2. **Summary of clean data statistics of metagenomic sequencing. **Table S3. **Assessment of metagenomic assembly. **Table S4. **Statistics of gene prediction based on assembled contigs. **Table S5. **List of genes involved in the nitrate reduction pathway. **Table S6. **List of genes involved in the (complete) nitrification pathway. **Table S7.** List of genes involved in the anammox pathway. **Table S8.** List of genes involved in the sulfate reduction pathway. **Table S9.** List of genes involved in the methanogenesis pathway. **Table S10.** List of genes involved in the methane oxidation pathway. **Table S11.** Relative abundance of chemotaxis-related genes obtained by metagenomic data. **Table S13. **Assessment of the “legacy → emergent stochasticity” PLS path model. **Fig. S1.** Age-depth model of the sediment profile **Fig. S2.** Principal coordinates analysis (PCoA) based on binary Jaccardand Bray-Curtis dissimilarities  **Fig. S3.** Error rate distributions in typical random forests for identification of damming-sensitive taxa. **Fig. S4.** Computation of the optimal breakpoints based on linear regression models for identification of damming-sensitive taxa. **Fig. S5.** The scaling coordinates of the proximity matrix from unsupervised random forest clustering. **Fig. S6. **Redundancy analysis (RDA) **Fig. S7.** Sigmoidal curve fitting for the abundance pattern of fermentation across depth. **Fig. S8.** Relationship between nearest taxon index (NTI) and depth using both individual and pooling sample sets. **Fig. S9.** Evidence from the phylogenetic-info-based null model that shows the deterministic assembly of the sediment microbial metacommunity. **Fig. S10.** A pattern of mean Levin’s niche breadth index of local communities at different depth layers.  **Fig. S11. **Coefficients of variation (CVs) of moisture, TOC, and temperature over time at each sediment layer.

**Additional file 2:** **Table S12.** Fermentation-related functional annotation of prokaryotic taxa based on FAPROTAX.

## Data Availability

The data generated in the current study are publicly available. The amplicon sequence files have been deposited in the NCBI Sequence Read Archive database (SRA) under accession number SRP154610 (BioProject PRJNA482178). The metagenomic sequence files are available at the NCBI SRA as part of BioProject PRJNA838605.
