# Supplementary material for: Anthropogenic activities mediate stratification and stability of microbial communities in freshwater sediments
Source: Microbiome. 2023 Aug 26;11:191. doi: 10.1186/s40168-023-01612-z (PMC10464086; doi:10.1186/s40168-023-01612-z)
Supplement: Supplementary file 2 — Additional file 1: Text S1. Introduction to the Chaohu Lake and damming history. Text S2. PCR amplification and 16S amplicon sequence processing. Text S3. Classification of environmental factors. Text S4. Prokaryotic motility and chemotaxis. Table S1. Sample summary and naming scheme. Table S2. Summary of clean data statistics of metagenomic sequencing. Table S3. Assessment of metagenomic assembly. Table S4. Statistics of gene prediction based on assembled contigs. Table S5. List of genes involved in the nitrate reduction pathway. Table S6. List of genes involved in the (complete) nitrification pathway. Table S7. List of genes involved in the anammox pathway. Table S8. List of genes involved in the sulfate reduction pathway. Table S9. List of genes involved in the methanogenesis pathway. Table S10. List of genes involved in the methane oxidation pathway. Table S11. Relative abundance of chemotaxis-related genes obtained by metagenomic data. Table S13. Assessment of the “legacy → emergent stochasticity” PLS path model. Fig. S1. Age-depth model of the sediment profile Fig. S2. Principal coordinates analysis (PCoA) based on binary Jaccardand Bray-Curtis dissimilarities Fig. S3. Error rate distributions in typical random forests for identification of damming-sensitive taxa. Fig. S4. Computation of the optimal breakpoints based on linear regression models for identification of damming-sensitive taxa. Fig. S5. The scaling coordinates of the proximity matrix from unsupervised random forest clustering. Fig. S6. Redundancy analysis (RDA) Fig. S7. Sigmoidal curve fitting for the abundance pattern of fermentation across depth. Fig. S8. Relationship between nearest taxon index (NTI) and depth using both individual and pooling sample sets. Fig. S9. Evidence from the phylogenetic-info-based null model that shows the deterministic assembly of the sediment microbial metacommunity. Fig. S10. A pattern of mean Levin’s niche breadth index of local communities at different depth layers [file 40168_2023_1612_MOESM1_ESM.docx]

**Supplementary Information**

**for**

**Anthropogenic activities mediate stratification and stability of microbial communities in freshwater sediments**

**Xiaotian Zhou^1,2^, Jay T. Lennon^3^, Xiang Lu^1,2^, Aidong Ruan^1,2*^**

*^1^ State Key Laboratory of Hydrology-Water Resources and Hydraulic Engineering, Nanjing 210098, China.*

*^2^ College of Hydrology and Water Resources,**Hohai University, Nanjing 210024, China.*

*^3^ Department of Biology, Indiana University, Bloomington, Indiana* *47405, USA*

*** *Corresponding author: Aidong Ruan (*[*adruan@hhu.edu.cn*](mailto:adruan@hhu.edu.cn)*)*

This file includes:

Supplementary Texts S1 to S4

Tables S1 to S11, Table S13

Figures. S1 to S11

References

**Text S1**

**Introduction to the Chaohu Lake and damming history**

Chaohu Lake (31°25′-31°43′ N, 117°16′-117°51′ E) is a natural impoundment located in the lower reach of the Yangtze River. It is a typical large shallow lake with an average depth of 3.0 m covering an area of 780 km^2^ [[1](#_ENREF_1)]. Chaohu Basin belongs to the northern subtropical monsoon climate zone with a mild climate. The region is characterized by moderate rainfall (annual precipitation ~1000 mm), distinct seasonality and a long frost-free period (~228 d/y) [[2](#_ENREF_2)]. Chaohu Lake and the Yangtze River are connected by the Yuxi River, the lake’s only outflow. Historically, during the rainy seasons, water from the Yangtze River would flow backward into Chaohu Lake, creating high water levels and the need for flood control. This motivated the construction of the Chaohu Dam on Yuxi River in 1962. Subsequent changes in hydrology combined with watershed development led to increased nutrient loading and symptoms of eutrophication [[3](#_ENREF_3)]. These transitions are well documented in lake sediments [[4](#_ENREF_4)], providing an opportunity to characterize the historical and contemporary factors that shape the sediment microbial community.

**Text S2**

**PCR amplification and 16S amplicon sequence processing**

PCR amplification was performed in a total volume of 50 μl, which contained 10 μl Buffer, 0.2 μl Q5 High-Fidelity DNA Polymerase, 10 μl High GC Enhancer, 1 μl dNTP, 10 μM of each primer and 60 ng genome DNA. PCR products were purified with E.Z.N.A.® Cycle Pure Kit (Omega), confirmed with gel electrophoresis and quantified by Quant-iT™ dsDNA HS Reagent. After the individual quantification step, amplicons were pooled by equal mass.

We prepared two sample treatments before 16S amplicon sequence processing: one kept individual samples separate (i.e., the 57 sediment subsamples), the other combined seasonal samples by pooling raw sequencing data of temporal samples of the same depth, which consisted of eleven depth-related samples. The merged samples were set to identify general vertical patterns without seasonal variation (see Fig. 1 and Table S1). The two sequencing datasets were processed separately as follows: FLASH v1.2.11 [[5](#_ENREF_5)] was used to merge pairs of reads from the original DNA fragments to produce raw tags (minimum overlap length 10bp). Quality trimming was performed using Trimmomatic v0.33 [[6](#_ENREF_6)] with a threshold of window size 50 and quality cutoff 20, and chimeric sequences were identified and removed in UCHIME v8.1 [[7](#_ENREF_7)]. Then, sequences were assigned to operational taxonomic units (OTUs) at 97% similarities using the UPARSE pipeline [[8](#_ENREF_8)]. We conducted an initial OTU filter with a threshold value of 0.005% [[9](#_ENREF_9)]. The SILVA Incremental Aligner (SINA v1.2.11) with the SILVA 132 database (<https://www.arb-silva.de>) was used for taxonomic classification [[10](#_ENREF_10)]. A phylogenetic tree was generated using FastTree2 [[11](#_ENREF_11)] after sequence alignment using the MAFFT software [[12](#_ENREF_12)]. The average effective sequence number of seasonal samples was 73668 ± 619 (mean ± SD), CV = 0.84%, and 390759 ± 56565, CV = 14 % for seasonal merged samples. To decrease biases of sequencing depth, OTU tables of each sample set were rarefied to the lowest number among samples as a standard number of sequences.

**Text S3**

**Classification of environmental factors**

We define present parameters as those context data derived from *in-situ* measurements, which indicates ongoing biological or chemical processes. The present parameters have unstable or transient features. Here, we serve pH, Conductivity, temperature, ORP and TOC as mainly present parameters.

Historical parameters are those conservative parameters reflecting the anthropogenic activities between 1950s and 1970s, which once introduced into the sediments will not change significantly and are therefore an expression of the lake’s history. Here, we serve damming and diatom-related eutrophication parameters as mainly conservative parameters.

Sedimentary parameters have both present and historical features. They are independent as a group because they typically reflect the effect of sediment age such as ongoing yet subtle changes from mineralization and burial processes. Specifically, D_50_ and MGS (mean grain size) provide insights into the sediment particle size, while total interstitial space (TIS) reflect the looseness of the sediment stacked structure.

**Text S4**

**Prokaryotic motility and chemotaxis**

Cell motility facilitates their flexibility and viability which allows organisms to contend with hydraulic fluctuations and steep environmental gradients [[13-17](#_ENREF_13)]. Motility has been reported to be important for collective behaviors, coexistence of species, and the maintenance of diversity [[16](#_ENREF_16)]. Constrained by the physical environment (e.g., fluid flow and porosity) and energy availability, sediment microbes are motile in shallower, porous, energy-rich sediments, whereas in deeper, compacted clay sediments, they tend to be motionlessly attached to solid particles [[14](#_ENREF_14), [18](#_ENREF_18)].

Chemotaxis is an important aspect by which cells sense chemical gradients and move directionally with preference. The oxycline of near-surface sediments is such a hotspot where microbes evolve various chemotactic strategies in response to O_2_ tension [[14](#_ENREF_14), [19-21](#_ENREF_19)]. From the perspective of community dynamics, chemotaxis reflects both active dispersal ability and environmental selection [[22](#_ENREF_22)]. It’s also the proxy of energy difference/gradient. The chemotaxis system usually consists of chemoreceptors (methyl-accepting chemotaxis proteins, MCPs) networked into cooperative arrays by coupling protein (*CheW*) and a two-component signaling kinase (*CheA*). Methyltransferase (*CheR*) and methylesterase (*CheB*) perform the methylation and de-methylation of the receptors to adapt to stimulus; *Ch*eY can bind to the flagellar motor when phosphorylated and thus change the rotation direction and move; CheA controls the two response regulators CheB and CheY [[23](#_ENREF_23), [24](#_ENREF_24)]. Such chemotaxis machinery structure and general features of excitation has been proved highly conserved among bacteria and archaea [[23-26](#_ENREF_23)]. Metagenomic sequencing enables us to estimate the relative abundance of chemotaxis genes in sediments at the community level which reflects natural selection during long-time burial. We estimated the vertical pattern of chemotaxis by the relative abundance of these prokaryotes-shared chemotaxis proteins obtained from metagenomic data (Table S11). Functional annotations of the metagenomic assembled scaftigs were performed to hit the KEGG database.

Chemotaxis works on the basis of the motility organelle flagellum that the taxis system relays environmental information to the flagellar motor to bias moving direction. However, unlike the chemotaxis system, archaeal flagella exhibit no obvious molecular similarity to bacterial flagella [[27-30](#_ENREF_27)]. Besides, different from the single flagellin found in most bacterial flagella, there are usually multiple flagellins in archaeal flagella. The distinct structure, assembly, spatial organization of flagellar, and distinct flagellar-assembly-related gene copy numbers between bacteria and archaea make it difficult to estimate the emergent motility intensity via abundance data predicted by metagenomic sequencing. For this reason, we estimated the metacommunity’s motility pattern across depth by digging information related to chemotaxis instead of flagellar assembly.

**Table S1.** Sample summary and naming scheme

| **Time**  **Series**  **Depth**  **(cm)** | | **Seasonal snapshots** | | | | | | | | **Burst shots** | | | |
| --- | --- | --- | --- | --- | --- | --- | --- | --- | --- | --- | --- | --- | --- |
|  |  | **Aug 2014** | **Oct 2014** | **Dec**  **2014** | **Mar 2015** | **May 2015** | **Jul 2015** | **Averaged**  **samples** | **Merged**  **samples** | **June 2019**  **H** | | | **Merged samples** |
|  |  | **A** | **B** | **C** | **D** | **E** | **F** |  |  | **a** | **b** | **c** |  |
| **2.5** | **01** | cA01 | cB01 | cC01 | cD01 | cE01 | cF01 | c01 | M01 | cH01a | cH01b | cH01c | cH01 |
| **7.5** | **02** | cA02 | cB02 | cC02 | cD02 | cE02 | cF02 | c02 | M02 | cH02a | cH02b | cH02c | cH02 |
| **12.5** | **03** | cA03 | cB03 | cC03 | cD03 | cE03 | cF03 | c03 | M03 | cH03a | cH03b | cH03c | cH03 |
| **17.5** | **04** | / | cB04 | cC04 | cD04 | cE04 | cF04 | c04 | M04 | cH04a | cH04b | cH04c | cH04 |
| **22.5** | **05** | cA05 | cB05 | / | cD05 | cE05 | cF05 | c05 | M05 | cH05a | cH05b | cH05c | cH05 |
| **27.5** | **06** | / | cB06 | cC06 | cD06 | cE06 | cF06 | c06 | M06 | cH06a | cH06b | cH06c | cH06 |
| **32.5** | **07** | cA07 | cB07 | cC07 | cD07 | cE07 | cF07 | c07 | M07 | cH07a | cH07b | cH07c | cH07 |
| **37.5** | **08** | / | cB08 | cC08 | cD08 | cE08 | cF08 | c08 | M08 | cH08a | cH08b | cH08c | cH08 |
| **42.5** | **09** | cA09 | / | cC09 | cD09 | cE09 | cF09 | c09 | M09 | cH09a | cH09b | cH09c | cH09 |
| **47.5** | **10** | / | / | cC10 | cD10 | cE10 | cF10 | c10 | M10 | cH10a | / | cH10c | cH10 |
| **52.5** | **11** | / | / | cC11 | cD11 | cE11 | cF11 | c11 | M11 | cH11a | / | cH11c | cH11 |

**Note:** the prefix “c” in all the individual sample names means the central part of the western Chaohu Lake. “/” means absence of the sample due to sampling depth or interval width.

**Table S2.** Summary of clean data statistics of metagenomic sequencing

| **Sample ID** | **Corresponding**  **layer** | **Clean data base**  (bp) | **Number of Reads** | **GC**  (%) | **Q20**  (%) | **Q30**  (%) |
| --- | --- | --- | --- | --- | --- | --- |
| CWS1 | 1 | 6314975210 | 20779920 | 58.43 | 98.22 | 95.06 |
| CWS2 | 2 | 7629039978 | 25064788 | 57.54 | 98.22 | 94.98 |
| CWS3 | 3 | 6036345032 | 19876863 | 56.76 | 98.24 | 94.98 |
| CWS4 | 4 | 6208517958 | 20451452 | 56.28 | 98.27 | 95.06 |
| CWS5 | 5 | 6513971086 | 21443830 | 55.05 | 98.32 | 95.20 |
| CWS6 | 6 | 6076423230 | 19991907 | 54.53 | 98.31 | 95.18 |
| CWS7 | 7 | 6501148672 | 21385641 | 53.54 | 98.3 | 95.15 |
| CWS8 | 8 | 6585589936 | 21654278 | 52.66 | 98.27 | 95.06 |
| CWS9 | 9 | 6492591858 | 21325855 | 53.05 | 98.24 | 94.97 |
| Average | / | 6484289218 | 21330504 | 55.32 | 98.27 | 95.07 |

**Table S3.** Assessment of metagenomic assembly

| **Sample ID** | **Corresponding**  **layer** | **Contig Num.** | **Total Len.**  (bp) | **Largest Len.**  (bp) | **N50**  (bp) | **GC**  (%) | **Mapped**  (%) |
| --- | --- | --- | --- | --- | --- | --- | --- |
| CWS1 | 1 | 456378 | 221195924 | 20223 | 470 | 59.59 | 31.17 |
| CWS2 | 2 | 541860 | 262716305 | 21765 | 466 | 58.01 | 31.26 |
| CWS3 | 3 | 408901 | 196414849 | 33802 | 463 | 57.36 | 27.61 |
| CWS4 | 4 | 449760 | 215521600 | 33122 | 461 | 56.75 | 30.16 |
| CWS5 | 5 | 488745 | 230849409 | 29797 | 454 | 54.96 | 30.43 |
| CWS6 | 6 | 500790 | 265913399 | 41721 | 511 | 53.96 | 38.52 |
| CWS7 | 7 | 587693 | 319234046 | 29709 | 522 | 53.23 | 42.71 |
| CWS8 | 8 | 587768 | 311416664 | 37544 | 508 | 52.08 | 40.5 |
| CWS9 | 9 | 601843 | 325715736 | 67288 | 522 | 52.87 | 43.34 |
| Average | / | 513749 | 260997548 | 34997 | 486 | 55.42 | 35.08 |

**Table S4.** Statistics of gene prediction based on assembled contigs

| **Sample ID** | **Layer** | **Gene Num.** | **Total Len.**  (bp) | **Average Len.**  (bp) | **Max. Len.**  (bp) | **Min. Len.**  (bp) | **Bacteria** | **Archaea** |
| --- | --- | --- | --- | --- | --- | --- | --- | --- |
| CWS1 | 1 | 512111 | 190660008 | 372 | 3768 | 102 | 0.87 | 0.01 |
| CWS2 | 2 | 605087 | 224950368 | 371 | 5814 | 102 | 0.85 | 0.03 |
| CWS3 | 3 | 454212 | 166730694 | 367 | 7695 | 102 | 0.82 | 0.05 |
| CWS4 | 4 | 496436 | 181556832 | 365 | 5016 | 102 | 0.78 | 0.09 |
| CWS5 | 5 | 531932 | 191798094 | 360 | 5004 | 102 | 0.72 | 0.14 |
| CWS6 | 6 | 564574 | 218067867 | 386 | 6606 | 102 | 0.66 | 0.20 |
| CWS7 | 7 | 669482 | 261653886 | 390 | 6129 | 102 | 0.62 | 0.22 |
| CWS8 | 8 | 664424 | 254732589 | 383 | 5430 | 102 | 0.58 | 0.24 |
| CWS9 | 9 | 686517 | 267274782 | 389 | 7581 | 102 | 0.57 | 0.23 |
| Average | / | 576086 | 217491680 | 376 | 5894 | 102 | 0.72 | 0.13 |

**Table S5.** List of genes involved in the nitrate reduction pathway (NCycDB)

| Class | Pathway | Gene (sub) families | Annotation | Relative abundance (*10^-5^) | | | | | | | | |
| --- | --- | --- | --- | --- | --- | --- | --- | --- | --- | --- | --- | --- |
|  |  |  |  | L1 | L2 | L3 | L4 | L5 | L6 | L7 | L8 | L9 |
| Assimilatory nitrate reduction | nitrate => ammonia | nirA | ferredoxin-nitrite reductase | 9.54 | 7.31 | 5.64 | 8.52 | 6.41 | 5.7 | 4.82 | 5.93 | 4.33 |
|  |  | narB | ferredoxin-nitrate reductase | 28.85 | 28.68 | 31.23 | 28.45 | 23.89 | 21.7 | 21.92 | 18.42 | 17.83 |
|  |  | nasA | assimilatory nitrate reductase catalytic subunit | 35.06 | 34.67 | 36.46 | 33.89 | 29.95 | 25.99 | 23.15 | 23.5 | 23.31 |
|  |  | NR | nitrate reductase (NAD(P)H) | 7.86 | 8.85 | 4.5 | 5.84 | 5.82 | 4.68 | 4.2 | 4.32 | 5.17 |
|  |  | NasB | assimilatory nitrate reductase electron transfer subunit | 11.61 | 14.36 | 9.82 | 10.35 | 8.56 | 6.93 | 6.86 | 5.32 | 4.05 |
|  |  | NarC | cytochrome b-561 | 10.63 | 11.56 | 9.5 | 8.32 | 12.94 | 10.09 | 10.33 | 5.73 | 6.23 |
| Dissimilatory nitrate reduction (NDRA) | nitrate => ammonia | nirB | nitrite reductase (NADH) large subunit | 39.71 | 38.27 | 36.96 | 39.49 | 26.82 | 24.72 | 22.22 | 24.74 | 27.44 |
|  |  | nirD | nitrite reductase (NADH) small subunit | 8.91 | 7.18 | 5.55 | 6.78 | 7 | 7.28 | 8.76 | 7.61 | 6.09 |
|  |  | nrfA | nitrite reductase (cytochrome c-552) | 19.31 | 19.18 | 15.87 | 15.59 | 15.99 | 12.38 | 13.66 | 10.05 | 9.05 |
|  |  | nrfB | cytochrome c-type protein NrfB | 1.13 | 0.19 | 0.18 | 0.89 | 0.23 | 0.18 | 0.15 | 0.06 | 0.06 |
|  |  | nrfC | protein NrfC | 97.65 | 94.25 | 97.57 | 92.34 | 70.17 | 72.76 | 63.47 | 53.53 | 52.62 |
|  |  | nrfD | protein NrfD | 3.79 | 4.73 | 3.14 | 4.46 | 3.67 | 4.54 | 3.12 | 3.73 | 2.79 |
| NDRA /Denitrification shared | nitrate => ammonia, nitrate => nitrogen | narG | nitrate reductase | 29.55 | 31.77 | 23.91 | 20.78 | 32.76 | 26.94 | 19.41 | 17.25 | 19.48 |
|  |  | narZ | nitrate reductase 2, alpha subunit | 22.24 | 17 | 18.64 | 17.33 | 9.62 | 4.96 | 5.61 | 3.03 | 4.58 |
|  |  | narH | nitrate reductase | 21.42 | 22.37 | 23.01 | 22.24 | 20.48 | 16.6 | 12.96 | 10.11 | 11.15 |
|  |  | narY | nitrate reductase 2, beta subunit | 2.46 | 2.22 | 2.86 | 1.58 | 1.17 | 0.91 | 1.4 | 1.32 | 1.37 |
|  |  | narJ | nitrate reductase molybdenum cofactor assembly chaperone | 13.06 | 8.11 | 10.87 | 11.16 | 6.33 | 3.83 | 4.67 | 3.06 | 3.58 |
|  |  | narV | nitrate reductase 2, gamma subunit | 0.51 | 0.29 | 0.09 | 0 | 0 | 0.14 | 0.09 | 0.29 | 0.17 |
|  |  | narI | nitrate reductase gamma subunit | 11.65 | 9.79 | 10 | 10.43 | 8.09 | 5.52 | 6.39 | 8.52 | 7.88 |
|  |  | narW | nitrate reductase 2, delta subunit | 0.9 | 0.61 | 1.14 | 0.57 | 0.16 | 0 | 0.2 | 0.12 | 0.25 |
|  |  | napA | periplasmic nitrate reductase NapA | 38.85 | 39.82 | 43.96 | 44.44 | 39.05 | 33.37 | 28.11 | 29.56 | 24.09 |
|  |  | napB | cytochrome c-type protein NapB | 4.77 | 4.73 | 2.05 | 4.06 | 2.5 | 1.05 | 1.58 | 1.7 | 3.61 |
|  |  | napC | cytochrome c-type protein NapC | 6.84 | 6.28 | 6.27 | 8.08 | 2.54 | 2.43 | 1.43 | 1.23 | 1.62 |
| Denitrification | nitrate => nitrogen | nirK | nitrite reductase (NO-forming) | 53.4 | 49.15 | 42.28 | 37.99 | 44.8 | 28.63 | 32 | 25 | 30.66 |
|  |  | nirS | nitrite reductase (NO-forming) / hydroxylamine reductase | 36.67 | 29.23 | 29.64 | 26.91 | 14.5 | 8.3 | 7.62 | 5.58 | 6.79 |
|  |  | norB | nitric oxide reductase subunit B | 27.83 | 29.39 | 31.46 | 27.84 | 20.05 | 17.79 | 16.29 | 15.57 | 16.01 |
|  |  | norC | nitric oxide reductase subunit C | 3.95 | 3.64 | 3.59 | 4.22 | 4.57 | 3.38 | 4.64 | 2.03 | 2.46 |
|  |  | nosZ | nitrous-oxide reductase | 36.82 | 35.22 | 31.33 | 26.83 | 23.73 | 18.71 | 15.47 | 14.98 | 14.22 |

**Table S6.** List of genes involved in the (complete) nitrification pathway (NCycDB)

| Class | Pathway | Gene (sub) families | Annotation | Relative abundance (*10^-5^) | | | | | | | | |
| --- | --- | --- | --- | --- | --- | --- | --- | --- | --- | --- | --- | --- |
|  |  |  |  | L1 | L2 | L3 | L4 | L5 | L6 | L7 | L8 | L9 |
| Nitrification | ammonia => nitrite => nitrate | nxrA | Nitrite oxidoreductase, alpha subunit | 0.23 | 0.32 | 0.09 | 0 | 0 | 0.18 | 0 | 0 | 0 |
|  |  | nxrB | Nitrite oxidoreductase, beta subunit | 1.17 | 2.12 | 1.41 | 0.69 | 3.24 | 0.74 | 1.2 | 0.97 | 0.31 |
| Nitrification/Complete nitrification | ammonia => nitrite, ammonia => nitrite => nitrate | amoA_A | Ammonia monooxygenase subunit A (archaea) | 1.56 | 0.39 | 0.64 | 0.2 | 1.88 | 0.46 | 0.96 | 0.5 | 0.98 |
|  |  | amoB_A | Ammonia monooxygenase subunit B (archaea) | 0.51 | 0.03 | 0 | 0 | 0.43 | 0.21 | 0.18 | 0.5 | 0.17 |
|  |  | amoC_A | Ammonia monooxygenase subunit C (archaea) | 0 | 0.13 | 0 | 0 | 0.43 | 0.18 | 0.2 | 0.21 | 0 |
|  |  | amoA_B | Ammonia monooxygenase subunit A (bacteria) | 1.21 | 1.77 | 2 | 0.93 | 0.63 | 0.14 | 0.55 | 0.53 | 0.7 |
|  |  | amoB_B | Ammonia monooxygenase subunit B (bacteria) | 0.98 | 0.8 | 1.68 | 0.61 | 0.39 | 0 | 0.12 | 0.21 | 0.2 |
|  |  | amoC_B | Ammonia monooxygenase subunit C (bacteria) | 1.45 | 1.77 | 1.73 | 1.01 | 0.39 | 0.21 | 0.18 | 0.09 | 0.42 |
|  |  | hao | Hydroxylamine dehydrogenase | 7.62 | 9.08 | 9.05 | 9.5 | 6.49 | 6.89 | 7.24 | 5.67 | 4.81 |

**Table S7.** List of genes involved in the anammox pathway (NCycDB)

| Class | Pathway | Gene families | Annotation | Relative abundance (*10^-5^) | | | | | | | | |
| --- | --- | --- | --- | --- | --- | --- | --- | --- | --- | --- | --- | --- |
|  |  |  |  | L1 | L2 | L3 | L4 | L5 | L6 | L7 | L8 | L9 |
| Anammox | Ammonia + nitrate/nitrite => nitrogen | hzsA | Hydrazine synthase subunit A | 0 | 0.13 | 0.23 | 0.37 | 0.16 | 0.14 | 0.18 | 0.12 | 0 |
|  |  | hzsB | Hydrazine synthase subunit B | 0.59 | 0.29 | 0.64 | 0.37 | 0.9 | 0.25 | 0.09 | 0.06 | 0.08 |
|  |  | hzsC | Hydrazine synthase subunit C | 0 | 0.32 | 0 | 0.12 | 0 | 0 | 0 | 0 | 0.11 |
|  |  | hzo | Hydrazine oxidoreductase | 0.35 | 0.19 | 0.73 | 0.24 | 0 | 0 | 0.18 | 0 | 0.25 |
|  |  | hdh | Hydrazine dehydrogenase | 0 | 0 | 0 | 0 | 0 | 0 | 0.06 | 0 | 0.14 |

**Table S8.** List of genes involved in the sulfate reduction pathway (KEGG)

| KEGG module | Class | Pathway | KO_ID | KO_name | KO_function | Relative abundance (*10^-5^) | | | | | | | | |
| --- | --- | --- | --- | --- | --- | --- | --- | --- | --- | --- | --- | --- | --- | --- |
|  |  |  |  |  |  | L1 | L2 | L3 | L4 | L5 | L6 | L7 | L8 | L9 |
| M00596 | Dissimilatory sulfate reduction (DSR) | sulfate => H_2_S | K00394 | aprA | adenylylsulfate reductase, subunit A | 24.7 | 20.96 | 30.01 | 22.93 | 19.27 | 14.14 | 9.87 | 7.02 | 5.51 |
|  |  |  | K00395 | aprB | adenylylsulfate reductase, subunit B | 8.99 | 7.85 | 8.18 | 8.32 | 8.01 | 3.97 | 2.36 | 2.14 | 1.76 |
|  |  |  | K11180 | dsrA | dissimilatory sulfite reductase alpha subunit | 14.93 | 15.19 | 16.14 | 16.88 | 12.63 | 9.95 | 7.59 | 8.78 | 6.04 |
|  |  |  | K11181 | dsrB | dissimilatory sulfite reductase beta subunit | 13.29 | 13.84 | 15.41 | 13.15 | 10.71 | 10.09 | 7.97 | 6.99 | 5.73 |
| M00176, M00596 | DSR/ASR shared | sulfate => H_2_S | K00958 | sat, met3 | sulfate adenylyltransferase | 22.2 | 24.21 | 24.14 | 21.71 | 21.5 | 18.46 | 13.46 | 13.19 | 10.4 |
| M00176 | Assimilatory sulfate reduction (ASR) | sulfate => H_2_S | K00390 | cysH | phosphoadenosine phosphosulfate reductase | 6.84 | 7.02 | 9.23 | 8.73 | 9.03 | 8.02 | 7.5 | 6.2 | 6.18 |
|  |  |  | K00392 | sir | sulfite reductase (ferredoxin) | 3.52 | 2.38 | 0.68 | 0.85 | 2.66 | 2.88 | 1.96 | 1.59 | 0.75 |
|  |  |  | K00380 | cysJ | sulfite reductase (NADPH) flavoprotein alpha-component | 1.68 | 2 | 1.23 | 0.77 | 0.23 | 0 | 0 | 0.09 | 0 |
|  |  |  | K00381 | cysI | sulfite reductase (NADPH) hemoprotein beta-component | 5.71 | 4.09 | 3.55 | 3.61 | 0.43 | 0.77 | 0.41 | 1.06 | 0.31 |
|  |  |  | K00955 | cysNC | bifunctional enzyme CysN/CysC | 24.9 | 17.29 | 19.14 | 13.72 | 8.8 | 3.48 | 2.89 | 1.47 | 1.12 |
|  |  |  | K00860 | cysC | adenylylsulfate kinase | 6.29 | 3.41 | 8.32 | 6.9 | 8.29 | 8.33 | 7.91 | 9.58 | 7.38 |
|  |  |  | K13811 | PAPSS | 3'-phosphoadenosine 5'-phosphosulfate synthase | 0 | 0 | 0.23 | 0.16 | 0.16 | 0 | 0 | 0 | 0.17 |

**Table S9.** List of genes involved in the methanogenesis pathway (KEGG)

| KEGG module | Pathway | KO_ID | KO_name | KO_function | Relative abundance (*10^-5^) | | | | | | | | |
| --- | --- | --- | --- | --- | --- | --- | --- | --- | --- | --- | --- | --- | --- |
|  |  |  |  |  | L1 | L2 | L3 | L4 | L5 | L6 | L7 | L8 | L9 |
| M00567, M00357, M00356, M00563 | All shared (CO2 => methane, acetate => methane,methanol => methane, methylamine/dimethylamine/trimethylamine => methane) | K00399 | mcrA | methyl-coenzyme M reductase alpha subunit | 0.82 | 2.48 | 3.82 | 5.6 | 5.24 | 4.15 | 1.87 | 1.88 | 1.56 |
|  |  | K00401 | mcrB | methyl-coenzyme M reductase beta subunit | 0.74 | 1.96 | 3.18 | 4.14 | 2.93 | 2.74 | 2.51 | 1.7 | 0.75 |
|  |  | K00402 | mcrG | methyl-coenzyme M reductase gamma subunit | 0.66 | 1.83 | 2.5 | 2.27 | 2.03 | 1.86 | 0.99 | 1 | 0.53 |
|  |  | K22480 | hdrA1 | heterodisulfide reductase subunit A1 | 0 | 0 | 0 | 0 | 0 | 0 | 0 | 0 | 0 |
|  |  | K22481 | hdrB1 | heterodisulfide reductase subunit B1 | 0 | 0 | 0 | 0 | 0 | 0 | 0 | 0 | 0 |
|  |  | K22482 | hdrC1 | heterodisulfide reductase subunit C1 | 0 | 0 | 0 | 0 | 0 | 0 | 0 | 0 | 0 |
|  |  | K03388 | hdrA2 | heterodisulfide reductase subunit A | 72.82 | 105.93 | 138.08 | 189.87 | 286.51 | 334.96 | 350.32 | 354.57 | 343.51 |
|  |  | K03389 | hdrB2 | heterodisulfide reductase subunit B | 25.96 | 35.31 | 42.33 | 46.92 | 61.49 | 77.86 | 81.01 | 79.51 | 81.27 |
|  |  | K03390 | hdrC2 | heterodisulfide reductase subunit C | 16.69 | 19.22 | 22.41 | 25.57 | 35.07 | 43.85 | 47.93 | 52 | 52.48 |
|  |  | K08264 | hdrD | heterodisulfide reductase subunit D | 0.94 | 1.67 | 0.95 | 2.48 | 1.17 | 2.6 | 1.58 | 1 | 0.98 |
|  |  | K08265 | hdrE | heterodisulfide reductase subunit E | 0 | 0.19 | 0.73 | 0.89 | 0 | 0.18 | 0.09 | 0.35 | 0.22 |
|  |  | K14126 | mvhA, vhuA, vhcA | F420-non-reducing hydrogenase large subunit | 19.74 | 22.92 | 23.19 | 33.16 | 41.91 | 46 | 49.37 | 51.86 | 52.4 |
|  |  | K14127 | mvhD, vhuD, vhcD | F420-non-reducing hydrogenase iron-sulfur subunit | 6.1 | 7.73 | 9.14 | 9.42 | 12.08 | 17.58 | 18.48 | 16.37 | 15.09 |
|  |  | K14128 | mvhG, vhuG, vhcG | F420-non-reducing hydrogenase small subunit | 9.15 | 13.13 | 14.32 | 19.28 | 24.16 | 31.79 | 34.56 | 33.26 | 30.1 |
|  |  | K00125 | fdhB | formate dehydrogenase (coenzyme F420) beta subunit | 3.28 | 7.76 | 9.96 | 9.5 | 8.68 | 9.81 | 9.08 | 7.99 | 8.44 |
|  |  | K22516 | fdhA | formate dehydrogenase (coenzyme F420) alpha subunit | 0 | 0 | 0 | 0 | 0 | 0 | 0 | 0 | 0 |
| M00356 | methanol => methane | K14080 | mtaA | [methyl-Co(III) methanol-specific corrinoid protein]:coenzyme M methyltransferase | 5.79 | 6.95 | 8.68 | 8.69 | 9.03 | 10.06 | 9.25 | 9.14 | 8.52 |
|  |  | K04480 | mtaB | methanol---5-hydroxybenzimidazolylcobamide Co-methyltransferase | 0.43 | 1 | 1 | 0.61 | 1.29 | 1.05 | 1.9 | 1.09 | 2.01 |
|  |  | K14081 | mtaC | methanol corrinoid protein | 0.27 | 0 | 0.27 | 0.2 | 0.23 | 0.11 | 0.26 | 0 | 0 |
| M00567 | CO2 => methane | K00200 | fwdA,fmdA | formylmethanofuran dehydrogenase subunit A | 4.1 | 4.06 | 7.77 | 9.9 | 12.39 | 16.99 | 19.47 | 21.92 | 20.68 |
|  |  | K00201 | fwdB,fmdB | formylmethanofuran dehydrogenase subunit B | 1.52 | 3.41 | 5.18 | 10.39 | 10.01 | 14.52 | 15.24 | 16.75 | 17.33 |
|  |  | K00202 | fwdC,fmdC | formylmethanofuran dehydrogenase subunit C | 1.49 | 1.77 | 2.77 | 6.17 | 5.43 | 9.28 | 9.72 | 11.58 | 10.79 |
|  |  | K00203 | fwdD,fmdD | formylmethanofuran dehydrogenase subunit D | 0.23 | 0.93 | 1.55 | 2.6 | 4.34 | 6.29 | 7.85 | 8.49 | 7.04 |
|  |  | K00205 | fwdF,fmdF | 4Fe-4S ferredoxin | 0.59 | 1.55 | 3 | 4.51 | 4.89 | 10.62 | 11.91 | 11.55 | 12.24 |
|  |  | K11261 | fwdE, fmdE | formylmethanofuran dehydrogenase subunit E | 3.6 | 4.89 | 5.27 | 5.84 | 5.98 | 10.16 | 9.4 | 7.11 | 6.62 |
|  |  | K11260 | fwdG | 4Fe-4S ferredoxin | 0.39 | 0.93 | 0.77 | 2.07 | 1.09 | 1.34 | 1.49 | 0.68 | 0.34 |
|  |  | K00204 | fwdH | 4Fe-4S ferredoxin | 0 | 0 | 0 | 0 | 0 | 0 | 0 | 0 | 0 |
|  |  | K00672 | ftr | formylmethanofuran--tetrahydromethanopterin N-formyltransferase | 1.64 | 2.22 | 4.86 | 6.7 | 9.97 | 18.04 | 20.14 | 23.95 | 21.52 |
|  |  | K01499 | mch | methenyltetrahydromethanopterin cyclohydrolase | 1.21 | 1.58 | 2.14 | 3.77 | 4.81 | 10.69 | 11.27 | 12.02 | 12.6 |
|  |  | K00319 | mtd | methylenetetrahydromethanopterin dehydrogenase | 0.35 | 1.55 | 0.86 | 1.7 | 3.09 | 4.18 | 5.11 | 5.02 | 4.3 |
|  |  | K00320 | mer | 5,10-methylenetetrahydromethanopterin reductase | 1.68 | 2.38 | 1.86 | 5.52 | 11.1 | 19.41 | 18.57 | 20.13 | 21.02 |
|  |  | K13942 | hmd | 5,10-methenyltetrahydromethanopterin hydrogenase | 0 | 0 | 0 | 0 | 0 | 0 | 0 | 0 | 0 |
| M00567, M00357 | CO2 => methane, acetate => methane | K00577 | mtrA | tetrahydromethanopterin S-methyltransferase subunit A | 1.76 | 4.28 | 7.23 | 7.1 | 4.93 | 5.13 | 6.04 | 4.2 | 4.5 |
|  |  | K00578 | mtrB | tetrahydromethanopterin S-methyltransferase subunit B | 0 | 0.71 | 1.32 | 1.18 | 0.2 | 1.13 | 0.67 | 0.73 | 0.34 |
|  |  | K00579 | mtrC | tetrahydromethanopterin S-methyltransferase subunit C | 0.04 | 0.42 | 1.59 | 1.79 | 0.86 | 1.65 | 0.73 | 0.62 | 0.34 |
|  |  | K00580 | mtrD | tetrahydromethanopterin S-methyltransferase subunit D | 0.43 | 0.87 | 0.82 | 2.39 | 0.98 | 1.62 | 0.73 | 0.56 | 0.42 |
|  |  | K00581 | mtrE | tetrahydromethanopterin S-methyltransferase subunit E | 0.55 | 1.29 | 0.86 | 2.07 | 1.25 | 0.91 | 0.73 | 0.88 | 0.31 |
|  |  | K00582 | mtrF | tetrahydromethanopterin S-methyltransferase subunit F | 0 | 0.52 | 1 | 0.77 | 0.7 | 0.63 | 0.5 | 0.41 | 0.28 |
|  |  | K00583 | mtrG | tetrahydromethanopterin S-methyltransferase subunit G | 0 | 0.19 | 0 | 0 | 0 | 0 | 0.23 | 0 | 0 |
|  |  | K00584 | mtrH | tetrahydromethanopterin S-methyltransferase subunit H | 2.58 | 4.47 | 5.5 | 6.74 | 7.78 | 10.27 | 11.18 | 12.37 | 12.55 |
| M00357 | acetate => methane | K00925* | ackA | acetate kinase | 26.93 | 25.69 | 22.28 | 23.09 | 16.18 | 10.69 | 9.98 | 8.58 | 8.72 |
|  |  | K00625* | E2.3.1.8, pta | phosphate acetyltransferase | 14.46 | 9.69 | 12.09 | 14.25 | 6.1 | 4.36 | 4.12 | 2.91 | 4.44 |
|  |  | K01895* | ACSS1_2, acs | acetyl-CoA synthetase | 111.48 | 115.43 | 118.44 | 110.68 | 99.3 | 91.22 | 75.61 | 77.07 | 75.4 |
|  |  | K00193 | cdhC | acetyl-CoA decarbonylase/synthase complex subunit beta | 0.27 | 2.77 | 4.73 | 8.08 | 13.84 | 16.14 | 19 | 16.19 | 16.91 |
|  |  | K00194 | cdhD | acetyl-CoA decarbonylase/synthase complex subunit delta | 10.2 | 10.53 | 13 | 12.1 | 21.19 | 27.04 | 27.67 | 28.38 | 28.39 |
|  |  | K00195 | cdhB | acetyl-CoA decarbonylase/synthase complex subunit epsilon | 14.54 | 19.7 | 20.87 | 22 | 28.42 | 32.85 | 36.78 | 35.84 | 35.55 |
| M00563 | methylamine/dimethylamine/trimethylamine => methane | K14082 | mtbA | [methyl-Co(III) methylamine-specific corrinoid protein]:coenzyme M methyltransferase | 0.31 | 0.45 | 0.14 | 0.32 | 0.39 | 0.56 | 0.82 | 0.82 | 0.42 |
|  |  | K14083 | mttB | trimethylamine---corrinoid protein Co-methyltransferase | 44.72 | 57.62 | 53.88 | 52.56 | 77.76 | 83.41 | 90.12 | 94.93 | 87.05 |
|  |  | K14084 | mttC | trimethylamine corrinoid protein | 1.56 | 1.22 | 2.18 | 3.08 | 3.48 | 4.92 | 7.21 | 6.32 | 6.93 |
|  |  | K16176 | mtmB | methylamine---corrinoid protein Co-methyltransferase | 1.37 | 2.32 | 2.82 | 4.71 | 5.63 | 9.78 | 12.49 | 9.31 | 8.83 |
|  |  | K16177 | mtmC | monomethylamine corrinoid protein | 0 | 0 | 0.41 | 0.08 | 0.31 | 0.46 | 0.35 | 0.41 | 0.45 |
|  |  | K16178 | mtbB | dimethylamine---corrinoid protein Co-methyltransferase | 0.51 | 0.74 | 0.91 | 0.73 | 1.21 | 0.53 | 1.49 | 1.35 | 1.03 |
|  |  | K16179 | mtbC | dimethylamine corrinoid protein | 0.27 | 0.39 | 0.14 | 1.14 | 2.42 | 4.85 | 4.52 | 4.47 | 2.96 |

Note: K00925, K00625, and K01895 are not methanogenesis-specific KOs; they are also involved in carbonhydrate metabolism (e.g., glycolysis/gluconeogenesis, pyruvate, and propanoate metabolism) and carbon fixation pathways. Thus, they were excluded when estimating the total abundance of methanogenesis.

**Table S10.** List of genes involved in the methane oxidation pathway (KEGG+NCycDB)

| KEGG Module | Pathway | KO_ID | KO_name | Annotation | Relative abundance (*10^-5^) | | | | | | | | |
| --- | --- | --- | --- | --- | --- | --- | --- | --- | --- | --- | --- | --- | --- |
|  |  |  |  |  | L1 | L2 | L3 | L4 | L5 | L6 | L7 | L8 | L9 |
| M00174 | methane => formaldehyde | K10944* | pmoA-amoA | methane/ammonia monooxygenase subunit A | 1.95 | 1.8 | 1.95 | 0.93 | 0.82 | 0.14 | 0.2 | 0.18 | 0.11 |
|  |  | K10945* | pmoB-amoB | methane/ammonia monooxygenase subunit B | 2.35 | 1.58 | 2.27 | 1.79 | 1.13 | 0.42 | 0.32 | 0.21 | 0 |
|  |  | K10946* | pmoC-amoC | methane/ammonia monooxygenase subunit C | 2.78 | 2.86 | 2.59 | 1.99 | 1.06 | 0.46 | 0.2 | 0.29 | 0.14 |
|  |  | K16157 | mmoX | methane monooxygenase component A alpha chain | 0 | 0 | 0 | 0 | 0 | 0 | 0 | 0 | 0 |
|  |  | K16158 | mmoY | methane monooxygenase component A beta chain | 0 | 0 | 0 | 0 | 0 | 0 | 0 | 0 | 0 |
|  |  | K16159 | mmoZ | methane monooxygenase component A gamma chain | 0 | 0 | 0 | 0 | 0 | 0 | 0 | 0 | 0 |
|  |  | K16160 | mmoB | methane monooxygenase regulatory protein B | 0 | 0 | 0 | 0 | 0 | 0 | 0 | 0 | 0 |
|  |  | K16161 | mmoC | methane monooxygenase component C | 0 | 0 | 0 | 0 | 0 | 0 | 0 | 0 | 0 |
|  |  | K16162 | mmoD | methane monooxygenase component D | 0 | 0 | 0 | 0 | 0 | 0 | 0 | 0 | 0 |
|  |  | K14028 | mdh1,mxaF | methanol dehydrogenase (cytochrome c) subunit 1 | 0 | 0 | 0 | 0 | 0 | 0 | 0 | 0 | 0 |
|  |  | K14029 | mdh2,mxaI | methanol dehydrogenase (cytochrome c) subunit 2 | 0 | 0 | 0 | 0 | 0 | 0 | 0 | 0 | 0 |
|  |  | K23995 | xoxF | lanthanide-dependent methanol dehydrogenase | 0 | 0 | 0 | 0 | 0 | 0 | 0 | 0 | 0 |
| NCycDB | | | pmoA | Particulate methane monooxygenase subunit A | 1.49 | 1.96 | 1.23 | 1.5 | 0.82 | 0.77 | 1.05 | 1.35 | 0.89 |
|  |  |  | pmoB | Particulate methane monooxygenase subunit A | 1.92 | 1.16 | 1.36 | 1.46 | 0.51 | 0.28 | 0.26 | 0.09 | 0 |
|  |  |  | pmoC | Particulate methane monooxygenase subunit A | 1.45 | 1.71 | 1.23 | 1.06 | 0.55 | 0.11 | 0 | 0.12 | 0.28 |

Note: The gene families pmoABC and amoABC have vague annotations in KEGG. For this, we invoked the NCycDB to re-annotate the pmoABC.

**Table S11.** Relative abundance of chemotaxis-related genes obtained by metagenomic data (KEGG)

| Class | KO_ID | KO_function | Relative abundance (*10^-5^) | | | | | | | | |
| --- | --- | --- | --- | --- | --- | --- | --- | --- | --- | --- | --- |
|  |  |  | L1 | L2 | L3 | L4 | L5 | L6 | L7 | L8 | L9 |
| TCSPs | K03407 | sensor kinase *CheA* | 8.33 | 10.04 | 8.91 | 11.45 | 8.91 | 7.7 | 7.77 | 6.85 | 6.12 |
| TCSPs | K03408 | purine-binding chemotaxis protein *CheW* | 5.16 | 5.38 | 6.55 | 6.13 | 6.1 | 4.68 | 3.91 | 2.64 | 2.63 |
| TCSPs | K03409 | chemotaxis protein *CheX* | 0.39 | 0.1 | 0.23 | 0.37 | 0.63 | 0.56 | 1.14 | 7.64 | 1.37 |
| TCSPs | K03410 | chemotaxis protein *CheC* | 0.43 | 1.38 | 3.05 | 1.62 | 1.64 | 0.7 | 1.46 | 0.59 | 0.59 |
| TCSPs | K03411 | chemotaxis protein *CheD* | 0.98 | 0.97 | 1.82 | 2.96 | 1.06 | 0.81 | 0.93 | 1.09 | 1.29 |
| TCSPs | K03412 | response regulator *CheB* | 3.4 | 4.57 | 5.59 | 5.84 | 5.24 | 4.75 | 4.61 | 4.82 | 3.44 |
| TCSPs | K03413 | response regulator *CheY* | 8.64 | 9.46 | 10.68 | 10.35 | 9.34 | 6.68 | 5.49 | 5.26 | 4.67 |
| TCSPs | K03414 | chemotaxis protein *CheZ* | 0.24 | 0.32 | 0 | 0.2 | 0 | 0 | 0 | 0 | 0 |
| TCSPs | K03415 | response regulator *CheV* | 0.47 | 0.77 | 0 | 0 | 0 | 0.14 | 0 | 0.06 | 0 |
| TCSPs | K00575 | chemotaxis protein methyltransferase *CheR* | 5.63 | 6.66 | 9.23 | 11.2 | 9.26 | 6.44 | 6.71 | 4.23 | 5.48 |
| TCSPs | K13924 | *CheB*/*CheR* fusion protein *CheBR* | 20.72 | 20.38 | 18.28 | 13.19 | 11.45 | 11.25 | 9.55 | 7.46 | 8.13 |
| MCPs | K05874 | methyl-accepting chemotaxis protein I, serine sensor receptor *tsr* | 0 | 0 | 0.14 | 0 | 0.59 | 0.42 | 0.32 | 0.21 | 0.28 |
| MCPs | K05875 | methyl-accepting chemotaxis protein II, aspartate sensor receptor *tar* | 0.27 | 0.23 | 0.5 | 0.16 | 0 | 0.14 | 0 | 0 | 0 |
| MCPs | K03406 | methyl-accepting chemotaxis protein *mcp* | 10.36 | 17.58 | 17.96 | 19.65 | 16.84 | 11.68 | 9.49 | 9.61 | 6.09 |
| MCPs | K03776 | aerotaxis receptor *aer* | 0.31 | 0.26 | 0.32 | 0.12 | 0 | 0 | 0 | 0 | 0 |

Note: TCSPs, two-component system proteins; MCPs, methyl-accepting chemotaxis proteins

**Table S13.** Assessment of the “legacy → emergent stochasticity” PLS path model

| **Measurement model (outer model) assessment** | | | | | | | | |
| --- | --- | --- | --- | --- | --- | --- | --- | --- |
| **Blocks** | **Mode** | **Num. of Indicators** | **Unidimensionality of indicators** | | | | **Loadings**  (Acceptable when >0.7) | **Crossloading rule** |
|  |  |  | **Cronbach's alpha**  (Acceptable when >0.7) | **Dillon-Goldstein's rho**  (Acceptable when >0.7) | **First eigenvalue**  (Acceptable when >1) | **Second eigenvalue** (Acceptable when <1) |  |  |
| Legacy effect | Formative | 3 | / | / | / | / | / | / |
| Environmental fluctuation | Formative | 2 | / | / | / | / | / | / |
| Energy difference | Reflective | 3 | 0.88 | 0.92 | 2.41 | 0.36 | 0.92/0.91/0.86 | Follow |
| Mean niche breadth | Reflective | 1 | 1 | 1 | 1 | 0 | 1 | Follow |
| Community fluctuation | Reflective | 1 | 1 | 1 | 1 | 0 | 1 | Follow |
| Emergent stochasticity | Reflective | 1 | 1 | 1 | 1 | 0 | 1 | Follow |
| **Structural model (inner model) assessment** | | | | | **Global criterion** | | | |
| **Name of latent variables** | **Type** | **R^2^ determination coefficients**  (Low: R^2^ < 0.30, Moderate: 0.30< R^2^ < 0.60, High: R^2^ > 0.60) | | **Mean redundancy** | **Goodness of Fit (GoF)**  (Acceptable when GoF > 0.7) | | | **If acceptable** |
| Energy difference | Endogenous | 0.67 | | 0.54 | 0.71 | | | YES |
| Mean niche breadth | Endogenous | 0.53 | | 0.53 |  |  |  |  |
| Community fluctuation | Endogenous | 0.94 | | 0.94 |  |  |  |  |
| Emergent stochasticity | Endogenous | 0.68 | | 0.68 |  |  |  |  |
| Legacy effect | Exogenous | / | | / |  |  |  |  |
| Environmental fluctuation | Exogenous | / | | / |  |  |  |  |

Note: R^2^ determination coefficients indicates the amount of variance in the endogenous latent variable explained by its independent latent variables.


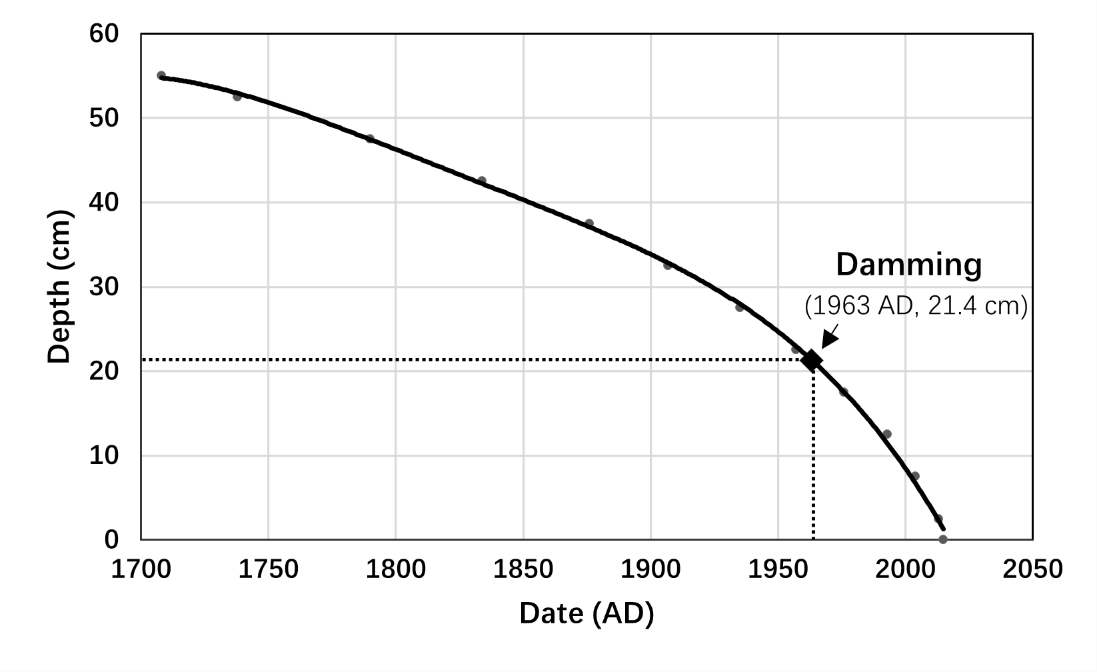


**Fig. S1** Age-depth model of the sediment profile extrapolated from dating model for core C1 in Chen et al. [[3](#_ENREF_3)] and core C4 in Zan et al.[[31](#_ENREF_31)]. Damming event is labeled by a diamond-shaped marker.


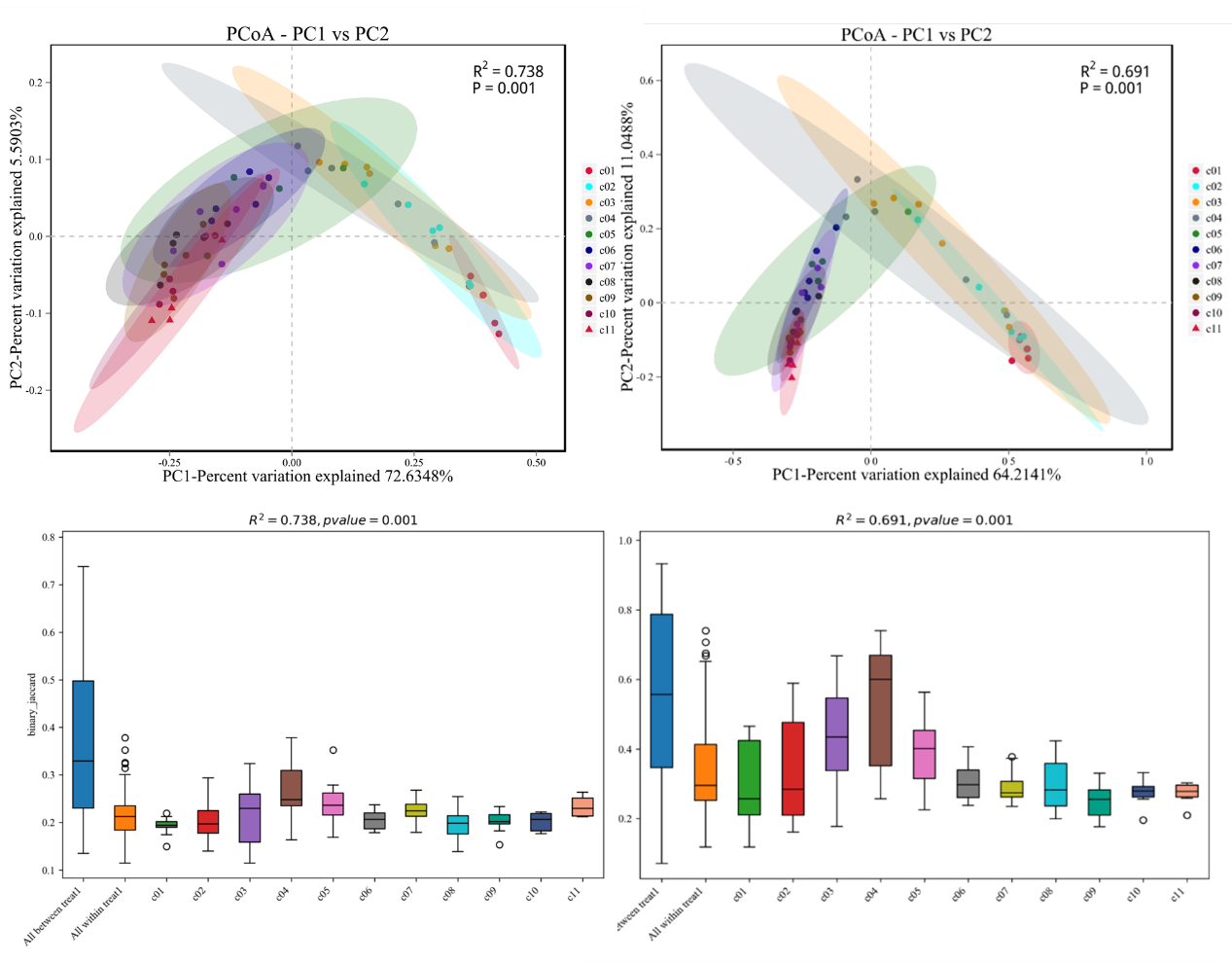


**Fig. S2** Principal coordinates analysis (PCoA) based on binary Jaccard (left two pics) and Bray-Curtis (right two pics) dissimilarities of OTUs illustrating continuous changes among bacterial communities grouped by depth. The size of 95% confidence circles shows within-group dissimilarities. The boxplots of beta-diversity between- and within- groups correspond to the PCoA results. PERMANOVA was conducted to estimate significant differences between groups. The abundance-based Bray-Curtis dissimilarity value is up to 0.95, implying almost a thorough turnover among communities across depth. The abundance-based dissimilarity among sediment horizons is much higher than that based on binary data, indicating species proportional composition variation rather than taxonomic turnover accounted for the beta diversity (i.e., abundance difference >> richness difference).


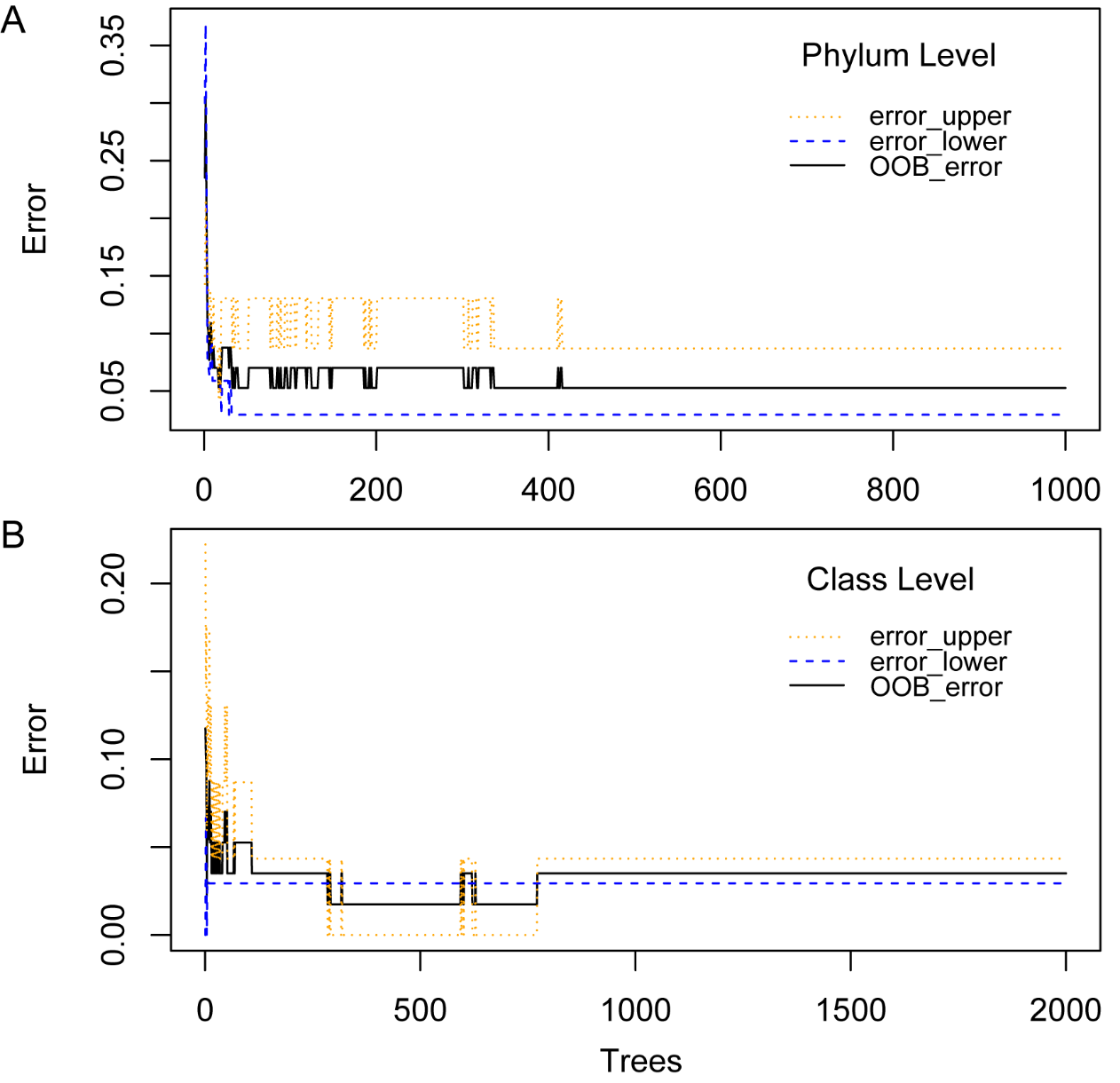


**Fig. S3** Error rate distributions in typical random forests for identification of damming-sensitive phyla (OOB error = 5.26%, ntree = 1000) and classes (OOB error = 3.51%, ntree = 2000).





**Fig. S4** Computation of the optimal breakpoints based on linear regression models for identification of damming-sensitive phyla (DSPs) and classes (DSCs). The first breakpoint (cluster I) gives the top features (DSPs: Bathyarchaeota, Spirochaetes, and Patescibacteria; DSCs: Dehalococcoidia, Bathyarchaeia, MBG-A, Spirochaetia, and Holophagae).


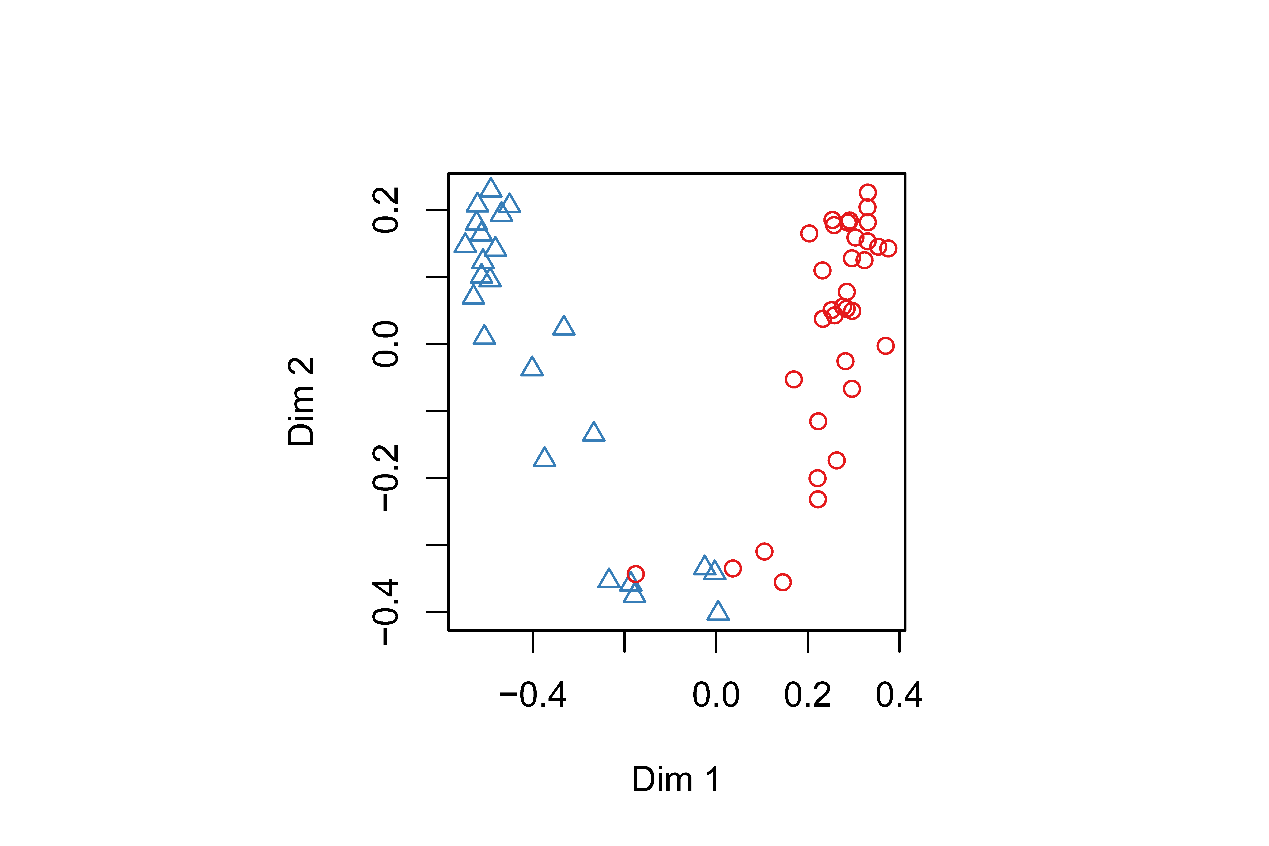


**Fig. S5** The scaling coordinates of the proximity matrix from unsupervised random forest clustering.


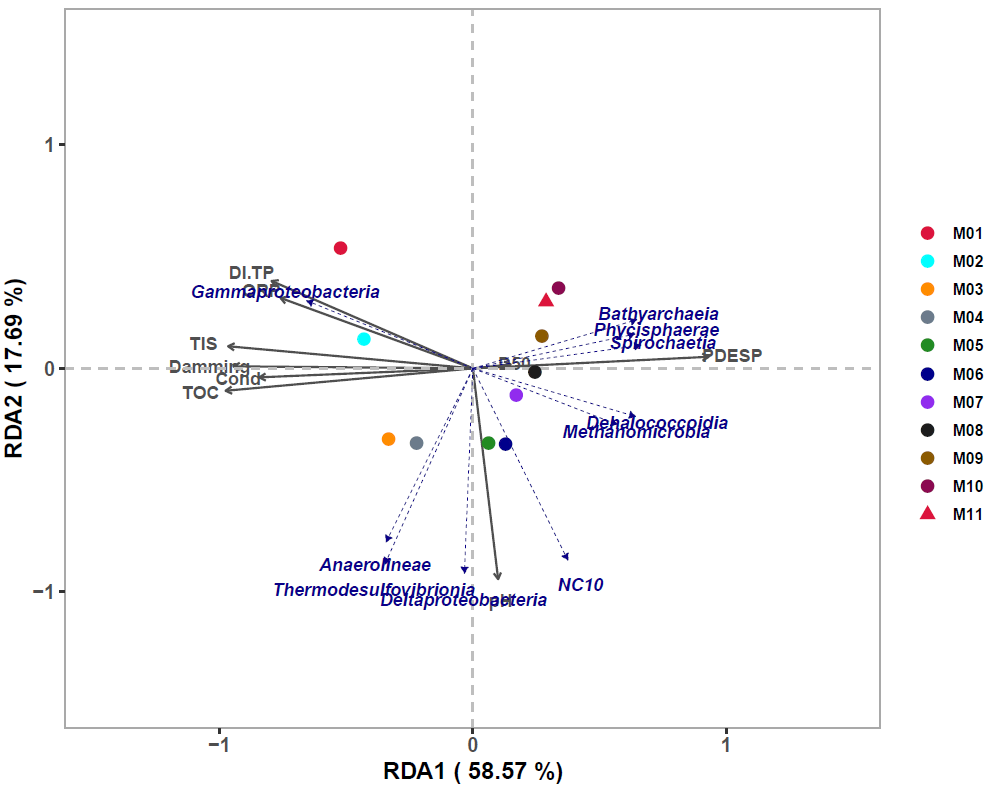


**Fig. S6** Redundancy analysis (RDA) among the top ten abundant classes (blue dotted arrow), environment variables, and eleven community samples.


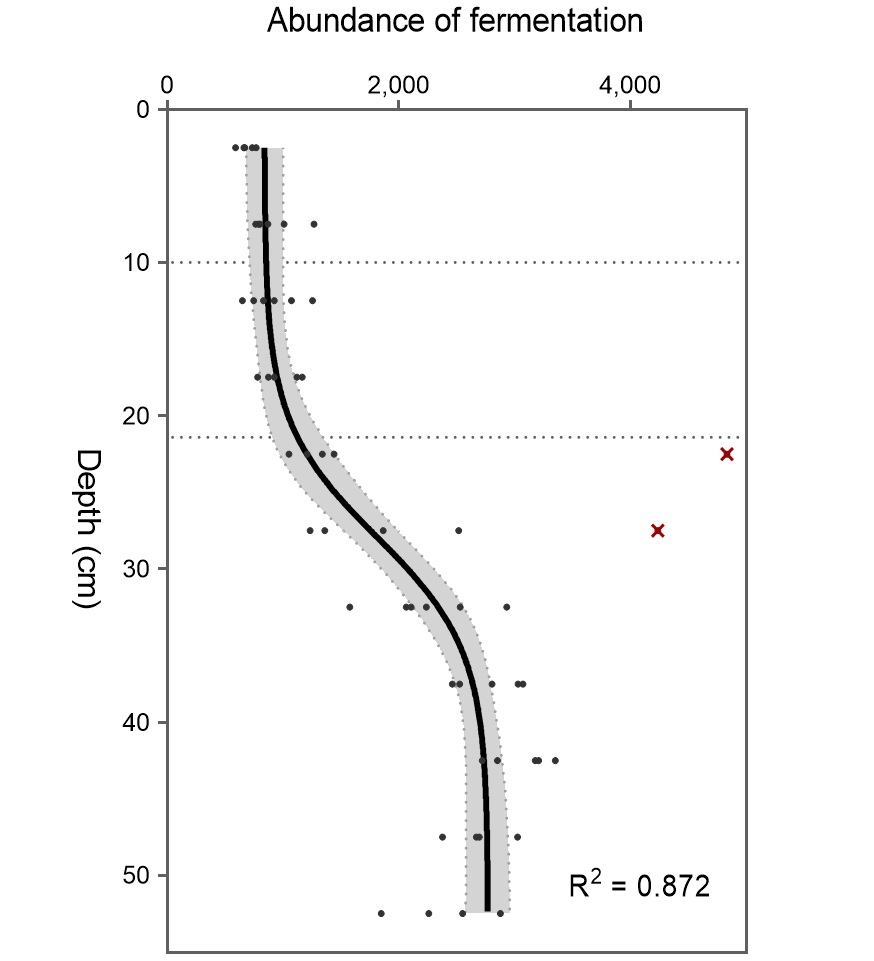


**Fig. S7** Sigmoidal curve fitting for the abundance pattern of fermentation across depth (R^2^ = 0.872, df = 51). Fermentation abundance was predicted against the FAPROTAX database using 16S rRNA data (see Table S12). Outliers are labeled by red X (excluded, Q = 1%). The grey area denotes 95% confidence bands.


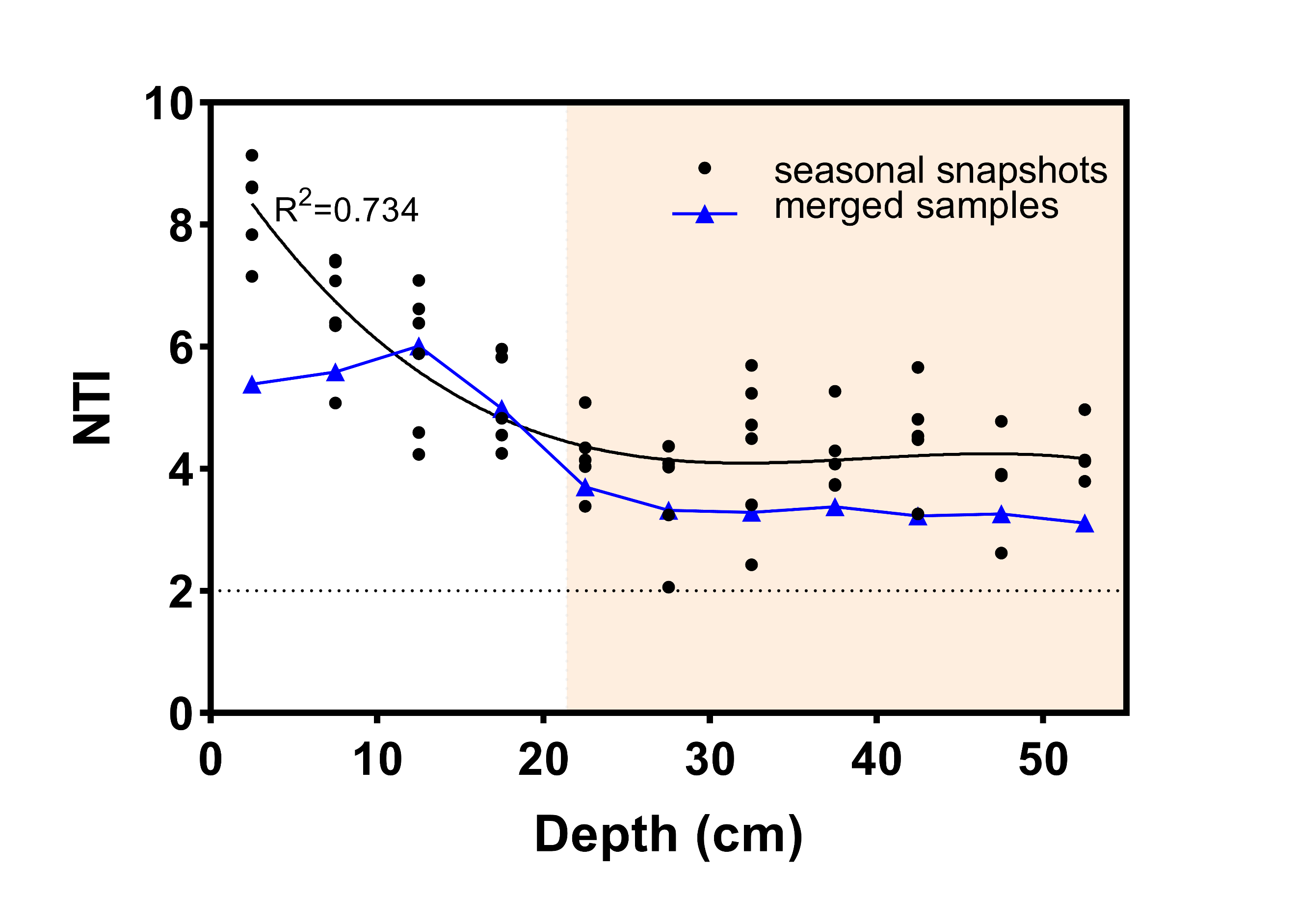


**Fig. S8** Relationship between nearest taxon index (NTI) and depth using both individual and pooling sample sets. Data of seasonal snapshots are fitted with third cubic polynomial model. NTI values below -2 or above +2 are considered statistically significant deviations that the observed MNTD (mean nearest taxon distance) is from the mean of the null distribution with 999 randomizations, which provide good evidence of deterministic environmental filtering for local community composition.


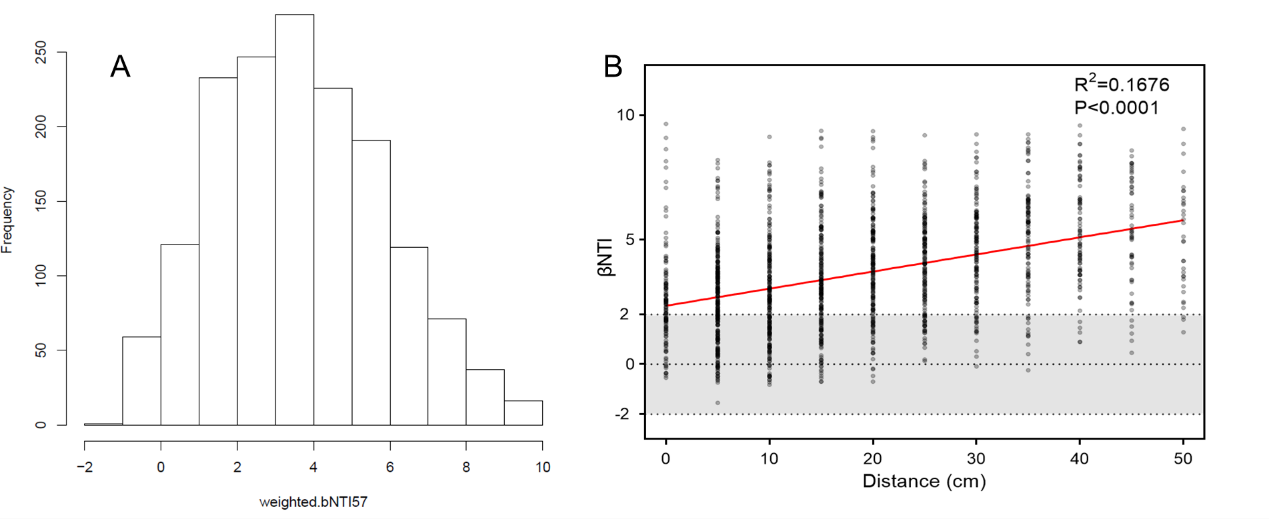


**Fig. S9** Evidence from the phylogenetic-info-based null model that shows the deterministic assembly of the sediment microbial metacommunity. **(A)** Histogram for distribution of abundance weighted βNTI values of the whole metacommunity. **(B)** Relationships between βNTI and distance of pairwise communities for all samples collected. The solid red line denotes a linear regression model indicating significant positive correlation between βNTI and spatial distance (P < 0.0001).

**Fig. S10** A pattern of mean Levin’s niche breadth index of local communities at different depth layers. The individual sample cC01 labeled in red is an outlier of the observed nonlinear trend (second-order polynomial fit).


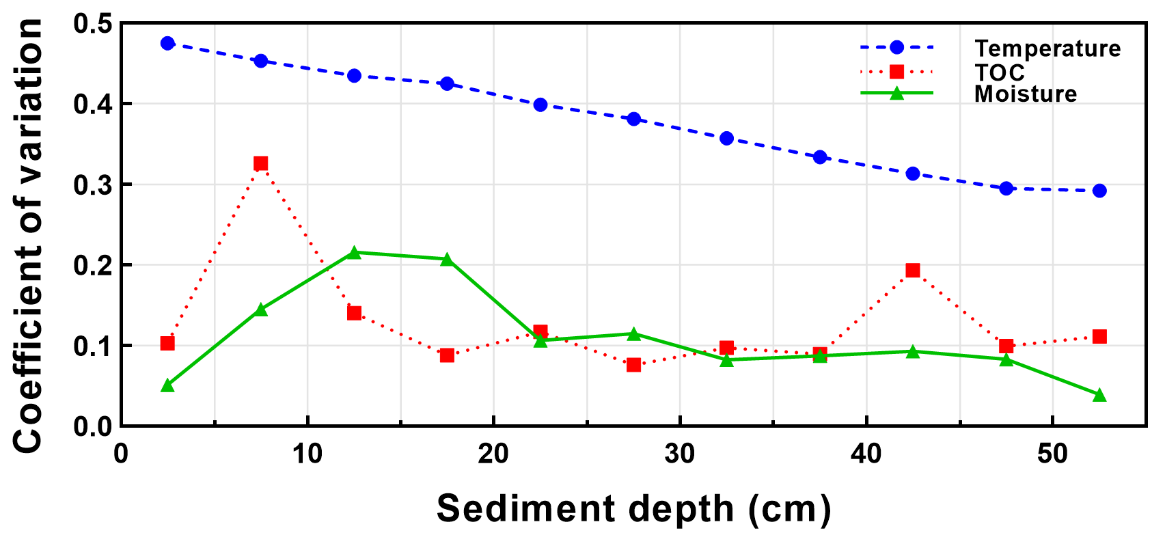


**Fig. S11** Coefficients of variation (CVs) of moisture (${Moi}_{(m)}$), TOC, and temperature over time at each sediment layer. ${CV}_{TOC}$ was excluded from indicator candidates of environmental fluctuation in PLS-PM, because i) it is not independent with *Δ_TOC_* of adjacent layers which reflects energy difference, ii) ${CV}_{TOC}$ gives low loading value in the outer model of environmental fluctuation.

**References**

1. Huo SL, Li CC, Xi BD, Yu ZQ, Yeager KM, Wu FC. Historical record of polychlorinated biphenyls (PCBs) and special occurrence of PCB 209 in a shallow fresh-water lake from eastern China. Chemosphere. 2017;184:832-40.

2. Liu E, Shen J, Birch GF, Yang X, Wu Y, Xue B. Human-induced change in sedimentary trace metals and phosphorus in Chaohu Lake, China, over the past half-millennium. J Paleolimnol. 2012;47(4):677-91.

3. Chen X, Yang XD, Dong XH, Liu QA. Nutrient dynamics linked to hydrological condition and anthropogenic nutrient loading in Chaohu Lake (southeast China). Hydrobiologia. 2011;661(1):223-34.

4. Sun KK, Chen X, Dong XH, Yang XD. Spatiotemporal patterns of carbon sequestration in a large shallow lake, Chaohu Lake: Evidence from multiple-core records. Limnologica. 2020;81.

5. Magoc T, Salzberg SL. FLASH: fast length adjustment of short reads to improve genome assemblies. Bioinformatics. 2011;27(21):2957-63.

6. Bolger AM, Lohse M, Usadel B. Trimmomatic: a flexible trimmer for Illumina sequence data. Bioinformatics. 2014;30(15):2114-20.

7. Edgar RC, Haas BJ, Clemente JC, Quince C, Knight R. UCHIME improves sensitivity and speed of chimera detection. Bioinformatics. 2011;27(16):2194-200.

8. Edgar RC. UPARSE: highly accurate OTU sequences from microbial amplicon reads. Nat Methods. 2013;10(10):996-8.

9. Bokulich NA, Subramanian S, Faith JJ, Gevers D, Gordon JI, Knight R, et al. Quality-filtering vastly improves diversity estimates from Illumina amplicon sequencing. Nat Methods. 2013;10(1):57-U11.

10. Pruesse E, Peplies J, Glockner FO. SINA: Accurate high-throughput multiple sequence alignment of ribosomal RNA genes. Bioinformatics. 2012;28(14):1823-9.

11. Price MN, Dehal PS, Arkin AP. FastTree 2-Approximately Maximum-Likelihood Trees for Large Alignments. Plos One. 2010;5(3).

12. Katoh K, Standley DM. MAFFT Multiple Sequence Alignment Software Version 7: Improvements in Performance and Usability. Molecular biology and evolution. 2013;30(4):772-80.

13. McCaulou DR, Bales RC, Arnold RG. Effect of Temperature-Controlled Motility on Transport of Bacteria and Microspheres Through Saturated Sediment. Water Resour Res. 1995;31(2):271-80.

14. Fenchel T. Motility of bacteria in sediments. Aquat Microb Ecol. 2008;51(1):23-30.

15. Tecon R, Or D. Biophysical processes supporting the diversity of microbial life in soil. Fems Microbiol Rev. 2017;41(5):599-623.

16. Gude S, Pinçe E, Taute KM, Seinen A-B, Shimizu TS, Tans SJ. Bacterial coexistence driven by motility and spatial competition. Nature. 2020;578(7796):588-92.

17. Mitchell JG, Kogure K. Bacterial motility: links to the environment and a driving force for microbial physics. FEMS microbiology ecology. 2006;55(1):3-16.

18. Son K, Brumley DR, Stocker R. Live from under the lens: exploring microbial motility with dynamic imaging and microfluidics. Nat Rev Microbiol. 2015;13(12):761-75.

19. Barbara GM, Mitchell JG. Formation of 30- to 40-micrometer-thick laminations by high-speed marine bacteria in microbial mats. Appl Environ Microbiol. 1996;62(11):3985-90.

20. Thar R, Fenchel T. Survey of Motile Microaerophilic Bacterial Morphotypes in the Oxygen Gradient above a Marine Sulfidic Sediment. Appl Environ Microb. 2005;71(7):3682-91.

21. Bjerg JT, Damgaard LR, Holm SA, Schramm A, Nielsen LP. Motility of Electric Cable Bacteria. Appl Environ Microb. 2016;82(13):3816-21.

22. Vellend M. Conceptual synthesis in community ecology. Q Rev Biol. 2010;85(2):183-206.

23. Briegel A, Ortega DR, Huang AN, Oikonomou CM, Gunsalus RP, Jensen GJ. Structural conservation of chemotaxis machinery across Archaea and Bacteria. Env Microbiol Rep. 2015;7(3):414-9.

24. Szurmant H, Ordal George W. Diversity in Chemotaxis Mechanisms among the Bacteria and Archaea. Microbiol Mol Biol R. 2004;68(2):301-19.

25. Salah Ud-Din AIM, Roujeinikova A. Methyl-accepting chemotaxis proteins: a core sensing element in prokaryotes and archaea. Cell Mol Life Sci. 2017;74(18):3293-303.

26. Ashby MK. Survey of the number of two-component response regulator genes in the complete and annotated genome sequences of prokaryotes. Fems Microbiol Lett. 2004;231(2):277-81.

27. Ng SY, Chaban B, Jarrell KF. Archaeal flagella, bacterial flagella and type IV pili: a comparison of genes and posttranslational modifications. J Mol Microbiol Biotechnol. 2006;11(3-5):167-91.

28. Jarrell KF, McBride MJ. The surprisingly diverse ways that prokaryotes move. Nat Rev Microbiol. 2008;6(6):466-76.

29. Thomas NA, Bardy SL, Jarrell KF. The archaeal flagellum: a different kind of prokaryotic motility structure. Fems Microbiol Rev. 2001;25(2):147-74.

30. Kreutzberger MAB, Sonani RR, Liu J, Chatterjee S, Wang F, Sebastian AL, et al. Convergent evolution in the supercoiling of prokaryotic flagellar filaments. Cell. 2022;185(19):3487-500.e14.

31. Zan FY, Huo SL, Xi BD, Zhu CW, Liao HQ, Zhang JT, et al. A 100-year sedimentary record of natural and anthropogenic impacts on a shallow eutrophic lake, Lake Chaohu, China. J Environ Monitor. 2012;14(3):804-16.
